# Intestinal microbial communities of rainbow trout (*Oncorhynchus mykiss*) may be improved by feeding a *Hermetia illucens* meal/low-fishmeal diet

**DOI:** 10.1007/s10695-020-00918-1

**Published:** 2021-01-03

**Authors:** Simona Rimoldi, Micaela Antonini, Laura Gasco, Federico Moroni, Genciana Terova

**Affiliations:** 1 Department of Biotechnology and Life Sciences, University of Insubria, Via J.H. Dunant, 3, 21100 Varese, Italy; 2 Department of Agricultural, Forest and Food Sciences, University of Turin, Largo P. Braccini 2, 10095 Grugliasco, Torino Italy

**Keywords:** Aquaculture, Intestinal microbiota, Metagenomics, Insect meal, *Hermetia illucens*, Rainbow trout

## Abstract

With demands and reliance on aquaculture still growing, there are various challenges to allow sustainable growth and the shift from fishmeal (FM) to other protein sources in aquafeed formulations is one of the most important. In this regard, interest in the use of insect meal (IM) in aquafeeds has grown rapidly. Accordingly, the aim of the present study was to assess the effects of dietary IM from *Hermetia illucens* (Hi) larvae included in a low-FM diet on gut microbial communities of rainbow trout (*Oncorhynchus mykiss*), in terms of both composition and function of microbiome. A feeding trial was conducted using 192 trout of about 100-g mean initial weight. Fish were fed in quadruplicate (4 tanks/diet) for 131 days with two diets: the control (Ctrl) contained 20% of FM as well as other protein sources, whereas the Hi diet contained 15% of Hi larvae meal to replace 50% of the FM contained in the Ctrl diet. High-throughput sequencing of 16S rRNA gene was used to identify the major feed and gut bacterial taxa, whereas Phylogenetic Investigation of Communities by Reconstruction of Unobserved States (PICRUSt) analysis was performed on gut bacterial genomes to identify the major active biological pathways. The inclusion of IM led to an increase in Firmicutes, mainly represented by Bacilli class and to a drastic reduction of Proteobacteria. Beneficial genera, such as *Lactobacillus* and *Bacillus*, were enriched in the gut of fish fed with the Hi diet, whereas the number of bacteria assigned to the pathogenic *Aeromonas* genus was drastically reduced in the same fish group. The metagenome functional data provided evidence that dietary IM inclusion can shape the metabolic activity of trout gut microbiota. In particular, intestinal microbiome of fish fed with IM may have the capacity to improve dietary carbohydrate utilization. Therefore, *H. illucens* meal is a promising protein source for trout nutrition, able to modulate gut microbial community by increasing the abundance of some bacteria taxa that are likely to play a key role in fish health.

**Supplementary Information:**

The online version contains supplementary material available at 10.1007/s10695-020-00918-1.

## Introduction

Aquaculture is growing rapidly and becoming integral in global food resources, supplying around half of the world’s seafood supply. One of the most important challenges that aquaculture sector is currently facing is the shift from fishmeal (FM) to other protein sources in aquafeed formulations and considerable efforts have been made so far to achieve this (Oliva-Teles et al. 2015). In this regard, interest in insect meals (IM) has grown rapidly within both scientific and fish farmer communities. 

The high potential of insects as an alternative protein source to substitute FM in aquafeeds is related to their nutritional value and life cycle process. Insects are rich in proteins (45–75% dry matter), essential amino acids, lipids, minerals, and vitamins, having a nutritional profile similar to FM (Gasco et al. 2020). Being a part of the natural diet of wild fish, insects have several ecological and economic advantages, too. They easily grow and reproduce on organic waste having a high substrate conversion efficiency. Furthermore, insect mass production generates low greenhouse gas and ammonia emissions thus meeting the recycling principles of the circular economy promoted by EU (van Huis and Oonincx 2017).

The EU Regulation No. 2017/893 (Annexe II of 24th May 2017) authorises the use in fish feeds of insect-derived proteins originating from seven species, namely, black soldier fly (*Hermetia illucens*), common housefly (*Musca domestica*), yellow mealworm (*Tenebrio molitor*), lesser mealworm (*Alphitobius diaperinus*), house cricket (*Acheta domesticus*), banded cricket (*Gryllodes sigillatus*) and field cricket (*Gryllus assimilis*).

Of these, black soldier fly (*Hermetia illucens*) is considered one of the most promising species to be used in feeds for salmonids, i.e. rainbow trout (*Oncorhynchus mykiss*) and Atlantic salmon (*Salmo salar*) (Henry et al. 2015; Renna et al. 2017; Belghit et al. 2018, 2019; Józefiak et al. 2019a; Li et al. 2020b; Fisher et al. 2020). High levels of dietary protein and lipid and low levels of carbohydrates are requested to meet the nutritional requirements of these fish species (Lock et al. 2018), and *H. illucens* (Hi) larvae satisfy these requirements as they contain a very high percentage of protein (36–48% DM) and fat (31–33% DM) and an essential amino acid profile similar to FM (Henry et al. 2015).

In the last years, a high number of scientific contributions on the use of IM in aquafeeds have been published demonstrating the great potential of Hi as a feed ingredient for cultured fish. The most recent evidences indicate that up to 50% of FM can be replaced by Hi larvae meal in Atlantic salmon and rainbow trout diet without any negative effect on growth performances or fillet quality (Renna et al. 2017; Bruni et al. 2018, 2020; Belghit et al. 2019).

In addition to the aforementioned nutrients, insects contain bioactive compounds that seem to have beneficial effects on animal health (Gasco et al. 2018). For instance, insects are rich in chitin and lauric acid that positively modulate host gut microbiota. Chitin is the primary constituent of the exoskeleton of arthropods, structurally analogous to cellulose, and therefore considered an insoluble fibre with potential prebiotic properties (Goycoolea et al. 2000). Lauric acid (C12:0), instead, is a medium-chain fatty acid (MCFA) known for its antimicrobial effects on Gram-positive bacteria (Spranghers et al. 2018).

However, few information is available about IM modulatory effect on fish intestinal microbiota (Parma et al. 2016; Bruni et al. 2018; Huyben et al. 2019; Belghit et al. 2019; Rimoldi et al. 2019; Terova et al. 2019; Józefiak et al. 2019a, b; Osimani et al. 2019; Li et al. 2020a). Indeed, only few studies have investigated the effect of dietary Hi meal inclusion on the gut bacterial communities of rainbow trout using high-throughput sequencing technologies (Huyben et al. 2019; Rimoldi et al. 2019; Terova et al. 2019).

The existing data suggest that fish gut microbiota is plastic and can be modulated by dietary insect meal that affects gut microbial diversity by enhancing the colonization of beneficial bacteria, such as lactic acid bacteria, which are widely used as probiotics in animal nutrition (Bruni et al. 2018; Rimoldi et al. 2019; Terova et al. 2019; Józefiak et al. 2019a, b). Such modulation of fish intestinal microbiota is reasonably expected since chitin, in addition to prebiotic properties, has antimicrobial and bacteriostatic effects on several harmful Gram-negative bacteria (Nawaz et al. 2018). Furthermore, the principal end products of chitin bacterial fermentation are short-chain fatty acids (SCFAs), such as acetate, propionate, and butyrate, which serve as the main energy sources for enterocytes.

Although the composition of fish intestinal bacterial community and the principles of its preservation are nearly known, we are still far from understanding how to manipulate gut microbiota through the diet to improve fish health. Intestinal microbiota, indeed, affects the immune response and digestive functions of the host through bacterial digestive enzyme production (Ghanbari et al. 2015). The commensal microorganisms can confer resistance by direct competition with pathogen for nutrients or may also produce bactericidal or bacteriostatic substances, such as lactic acid, hydrogen peroxide, bacteriocins, or biosurfactants (Corr et al. 2007; Gudiña et al. 2015).

Accordingly, the aim of the present study was to assess the effects of dietary inclusion of *H. illucens* larva meal as a replacer of FM on the gut microbial community of rainbow trout in terms of both microbiota’s composition and function. Furthermore, since previous studies of our group (Rimoldi et al. 2019; Terova et al. 2019) were focused on testing different inclusion levels of Hi in a high-FM diet, the aim of the present research was to investigate the inclusion of Hi in a practical (low FM) formulation context.

High-throughput sequencing of 16S rRNA gene was used to identify the dynamics of major gut bacterial taxa in response to diet. An in silico analysis through bioinformatics software package PICRUSt was performed on bacterial genomes to identify the major active biological pathways of gut bacteria.

## Materials and methods

### Ethics statement

The trial was conducted at the DISAFA Experimental Facility of the University of Turin (Italy). All procedures involving fish comply with the guidelines of the European Union Council (2010/63/EU) for the use and care of experimental animals. The Ethical Committee of the University of Turin (protocol no. 143811) approved the experimental protocol.

### Diets

Two diets were formulated to be isonitrogenous, isolipidic, and isoenergetic (Table 1). The first diet (control (Ctrl)) contained 20% of FM as well as other protein sources (wheat gluten, soybean meal, and haemoglobin), whereas the second diet (Hi15) contained 15% of *Hermetia illucens* (Hi) larva meal to replace 50% of the FM contained in the Ctrl diet. *Hermetia illucens* larva meal was provided by MUTATEC (Caumont-sur-Durance, France; https://mutatec.com/). Due to differences in chemical composition between Hi and FM and to ensure isonitrogenous, isolipidic, and isoenergetic diets, the level of inclusion of porcine haemoglobin and wheat starch slightly changed.

**Table 1:** Ingredients (g kg^−1^) and proximate composition of the experimental diets

Ingredients	Ctrl	Hi15
Fishmeal^a^	200.0	100.0
*Hermetia illucens* larva meal^b^	0.0	150.0
Wheat gluten	130.0	130.0
Soybean meal	200.0	200.0
Porcine haemoglobin	92.0	82.0
Wheat starch	233.9	193.9
Fish oil	69.8	69.8
Soybean oil	69.8	69.8
Minerals^c^	2.5	2.5
Vitamins^d^	2.0	2.0
Chemical analysis		
Dry matter (g 100 g^−1^)	97.15	96.56
Ash (g 100 g^−1^, as fed)	5.83	5.45
Crude protein (g 100 g^−1^, as fed)	45.60	46.14
Ether extract (g 100 g^−1^, as fed)	14.91	14.32
Gross energy (MJ kg^−1^, as fed)^e^	22.43	22.56

^a^Purchased from Corpesca S.A. (Santiago, Chile). ^b^Provided by MUTATEC, Caumont-sur-Durance, France (https://mutatec.com/). ^c^Mineral mixture: provided by Skretting. ^d^Vitamin mixture provided by Skretting. ^e^Determined by calorimetric bomb

Chemical analysis values are reported as mean of duplicate analyses

All feeds were prepared through cold pelleting at the experimental facility of the Department of Agricultural, Forest and Food Science (DISAFA) of the University of Turin (Torino, Italy). Briefly, all grounded ingredients were mixed with oil and desired consistency for pelleting was gained by adding water to the mixture. Each diet was cold pelleted using a 2.5-mm die meat grinder and the obtained pellet was dried at 50 °C for 48 h. Diets were stored in dark bags at a controlled temperature and humidity conditions.

### Feeding trial and fish sampling

A total of 192 rainbow trout with an initial mean body weight of about 100 g were randomly distributed in 8 outdoor fibre glass tanks of 0.4 m^3^ connected to a flow through an open system supplied with artesian well water (constant temperature of 13 ± 1 °C, 8 L min^−1^, DO 7.6–8.7 mg L^−1^). Fish were manually fed with two experimental diets in quadruplicate (four tanks/diet). The feeding rate was restricted to 1.4% of biomass for all the duration of the trial (131 days). Fish mortality was checked and recorded every day. At the end of the feeding trial, eight fish/dietary groups (2 fish/tank) were sacrificed by over anaesthesia with MS-222 (PHARMAQ Ltd., UK; 500 mg/L). The intestine was aseptically isolated from each fish, and the faecal matter was obtained by squeezing out and scrapping the intestinal mucosa with a sterile spatula, in order to collect both the digesta- and the mucosa-associated microbiota (transit and resident microbiota). The microbiota samples were then transferred into a sterile 2-mL tube containing 800 μL of Xpedition™ Lysis/Stabilization Solution (Zymo Research, Irvine, CA, USA) and then stored at room temperature, until DNA extraction (within 48 h).

### Bacterial DNA extraction from feeds and fish gut and 16S rRNA gene amplicon library construction

The amplification of the V4 region of the bacterial 16S rRNA gene and amplicon library construction were conducted as previously reported by our group (Rimoldi et al. 2018, 2019). In brief, DNeasy PowerSoil® Kit (Qiagen, Milan, Italy) was used to extract DNA from 250 mg of intestinal contents and from 200 mg of feed (3 replicates for each diet). The V4 hypervariable region of the 16S rRNA gene was amplified by PCR using forward primer 515F: 5′-GTGYCAGCMGCCGCGGTAA-3′ and reverse primer 806R: 5′-GGACTACNVGGGTWTCTAAT-3′. Amplicons were cleaned up followed by PCR to attach unique paired-end adapters with unique indices using Nextera XT Index Kit Library, in accordance with the Illumina protocol “16S Metagenomic Sequencing Library Preparation for Illumina MiSeq System” (#15044223 rev. B). Libraries were then quantified by qRT-PCR and pooled in one tube at equimolar concentrations. The amplicon library was pair-ended sequenced (2 × 250) on a MiSeq sequencing platform (Illumina). All sequences were submitted to European Nucleotide Archive (EBI ENA).

### Metabarcoding data analysis

The raw sequences were processed and analysed using QIIME™ 2 (v. 2018.4) at the default setting (Bolyen et al. 2019). The reads were trimmed at both 3′ and 5′ ends using Cutadapt v.2018.4.0 software, filtered for base quality (*Q* > 30), and merged. Filtered reads were dereplicated; singletons and chimeric sequences were removed using QIIME DADA2 denoise-paired command. All sequences were then clustered into operational taxonomic units (OTUs) at a 97% similarity cut-off. OTUs were classified using the reference Greengenes v. 13.8 as reference database (http://greengenes.lbl.gov/) down to genus level. Chloroplasts as well as sequences that were eukaryotic were removed. Sequences that had a frequency lower than 0.005% were removed from the dataset. Alpha rarefaction curves were plotted to determine the adequacy of sequencing depth. Alpha diversity indexes (Chao 1, observed OTUs, Shannon, Faith-PD, and evenness) were calculated to explain the species richness and diversity in each sample. Good’s coverage estimator was used to assess the percentage of the total species that are represented in a sample. Principal coordinates analysis (PCoA) was conducted to visualize similarities or dissimilarities of data based on unweighted UniFrac and weighted UniFrac distance metric (Lozupone and Knight 2005; Lozupone et al. 2007).

### Functional analysis of intestinal microbiota

Phylogenetic Investigation of Communities by Reconstruction of Unobserved States (PICRUSt) (Langille et al. 2013) was used to perform the predicted functional analysis (Langille et al. 2013). Taxonomic classification was performed using QIIME2 feature-classifier classify-sklearn function, a Naive Bayes classifier that was trained on the Greengenes v. 13.8 as reference database (http://greengenes.lbl.gov/) at 99% of similarity. The corresponding biom table was generated using the tools export function and used as input for the PICRUSt pipeline. In brief, PICRUSt was first used to correct biom tables for 16S rRNA copy numbers and subsequently used to predict KEGG (Kyoto Encyclopedia of Genes and Genomes) orthologues (KO). The maximum allowed Nearest Sequenced Taxon Index (NSTI) value was set to 2 to control for the overall accuracy of the metagenomic predictions. The output data generated with PICRUSt were subsequently uploaded to the Statistical Analysis of Metagenomic Profiles (STAMP) software package (Parks et al. 2014) for further downstream statistical analyses. A two-sided Welch *t* test with 95% confidence was applied to identify differences in microbial metabolic pathways between two groups.

### Statistical analysis

Normality and homogeneity of variance of data were checked by Shapiro-Wilk and Levene’s test, respectively. To test null hypothesis (*p* < 0.05), Student’s *t* test or nonparametric Mann-Whitney *U* test was applied depending on normality and homoscedasticity of the data. All analyses were performed using Past3 software (Hammer et al. 2001). To perform statistics on microbial relative abundance data, the percentage values were firstly angular transformed. Only those taxa with an overall abundance of more than 1% (up to order level) and 0.5% at family and genus levels were considered for the analysis. The significance of the calculated beta-diversity dissimilarities was assessed by nonparametric analysis of similarities (ANOSIM) and PERMANOVA tests based on 999 permutations using QIIME script “compare_categories.py”.

## Results

### Metabarcoding sequencing outcome

Sixteen intestinal and six feed samples were efficiently and correctly sequenced on an Illumina MiSeq platform. An overall sequences of 1,652,358 corresponding to an average of 75,107 ± 16,411 sequences per sample, was retained after the quality filtering and processing of sequencing reads.

Dataset was representative of bacterial communities due to Good’s coverage estimators for all samples that were greater than 99%.The sequencing depth was set based on the saturation phase of the alpha diversity rarefaction curves at 10,780 sequences in both feed and intestinal content samples (Supplementary Fig. 1, Online Resource 1). All sequencing data were submitted to the European Nucleotide Archive (EBI ENA) public database, under the accession code PRJEB38953.

### Characterization of feed-associated bacterial communities

**Fig. 1:**
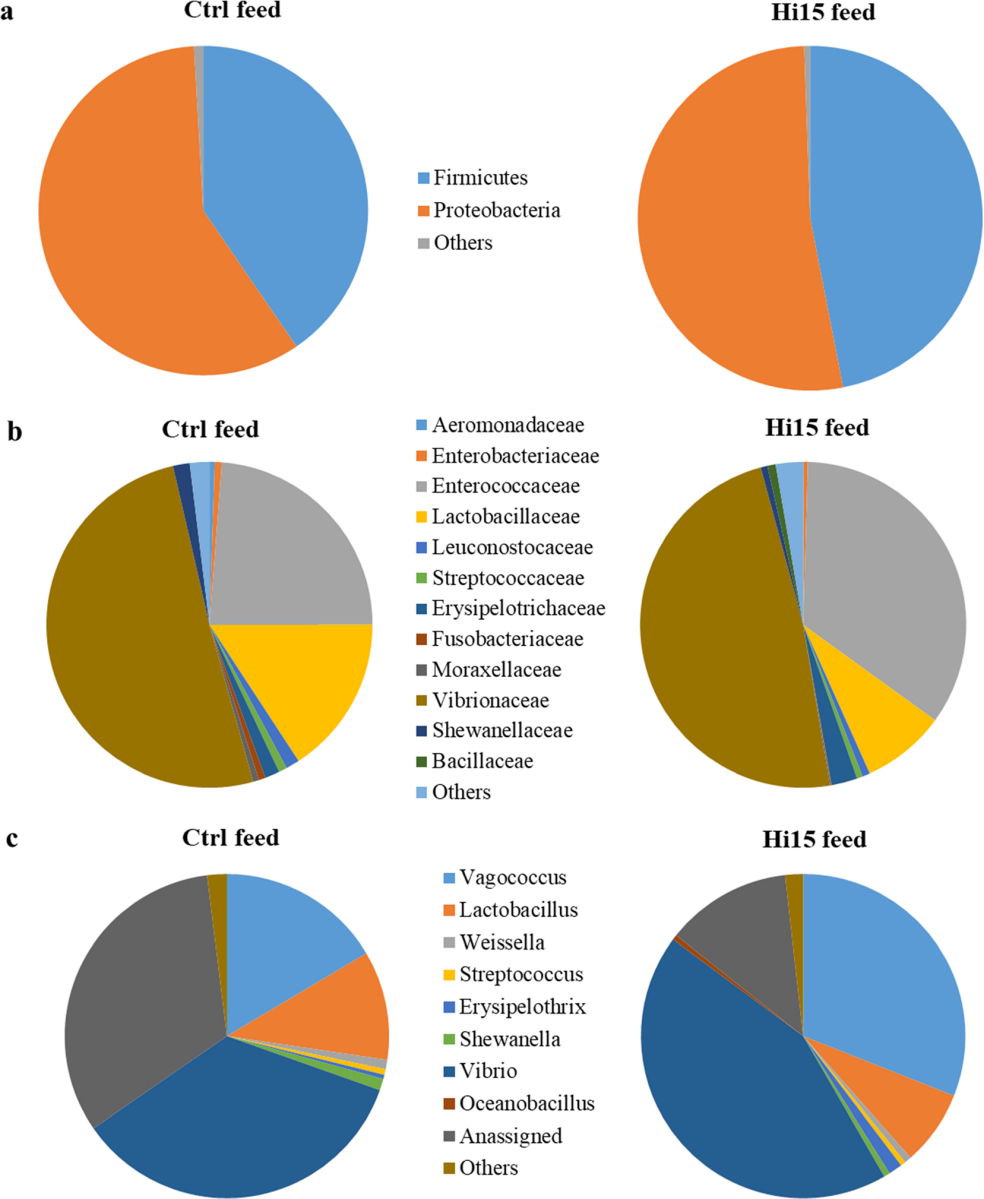
Relative abundance (%) of the most prevalent bacteria in Ctrl and feeds at phylum (**a**), family (**b**), and genus (**c**) taxonomic level. Only bacteria with an overall abundance of 0.5% were reported. Bacteria with lower abundance were pooled and indicated as “others”

A total of 38,073 and 34,672 high-quality reads was taxonomically classified for Ctrl and Hi15 feed samples, respectively. The high-throughput sequencing analysis revealed that the microbial profiles of feed were mainly comprised of 2 phyla, 4 classes, 6 orders, 12 families, and 8 genera. The most abundant taxa of bacteria at the phylum, family, and genus levels are shown in Fig. 1. The complete list of OTUs found in feeds with their relative abundances is given in Online Resource 2.

The microbial community diversity of feeds was evaluated by alpha diversity analysis, and indices are shown in Table 2. No differences in terms of species richness (Chao 1) and biodiversity (Shannon diversity index) or any other considered alpha diversity indexes were found between feed-associated communities. The relative abundances (%) of the most abundant taxa found in feed samples are listed in Supplementary Table 1 (Online Resource 3).

**Table 2:** Alpha diversity metrics (rarefied at 10,780 sequences) of feed microbial communities. All data are reported as mean values (*n* = 3) ± SD

Item	Ctrl feed	Hi15 feed	*p* value
Observed OTUs	340.67 ± 3.51	346.33 ± 9.24	0.48
Chao 1	368.91 ± 4.95	368.00 ± 21.28	0.94
Faith-PD	5.11 ± 0.32	5.60 ± 0.37	0.12
Shannon	6.00 ± 0.08	5.82 ± 0.06	0.05
Evenness	0.71 ± 0.01	0.69 ± 0.01	0.05

At phylum level, Hi15 feed was characterized by higher percentage of Firmicutes (47%), mainly represented by Bacilli class than the Ctrl feed (40%). Conversely, microbiota associated with Ctrl showed a higher relative abundance of Proteobacteria (58%), principally belonging to Alpha- and Gammaproteobacteria classes (Fig. 1a, Supplementary Table 1). Accordingly, a high amount of the Enterococcaceae (34%), Erysipelotrichaceae (2.5%), and Bacillaceae (0.8%) families was found in the diet with insect meal. Ctrl feed was instead rich in Lactobacillaceae (15.8%), Leuconostocaceae (1.3%), Fusobacteriaceae (0.7%), and Shewanellaceae (1.7%) (Fig. 1b; Supplementary Table 1). At genus level, Ctrl feed had higher relative abundance of *Lactobacillus*, *Weissella*, and *Shewanella* than Hi15 feed, which was instead rich in *Vagococcus*, *Erysipelothrix*, and *Vibrio* genera (Fig. 1c, Supplementary Table 1). Genus *Oceanobacillu*s was found only associated to insect-based feed.

### Microbial profile and dietary modulation of trout gut communities

**Fig. 2:**
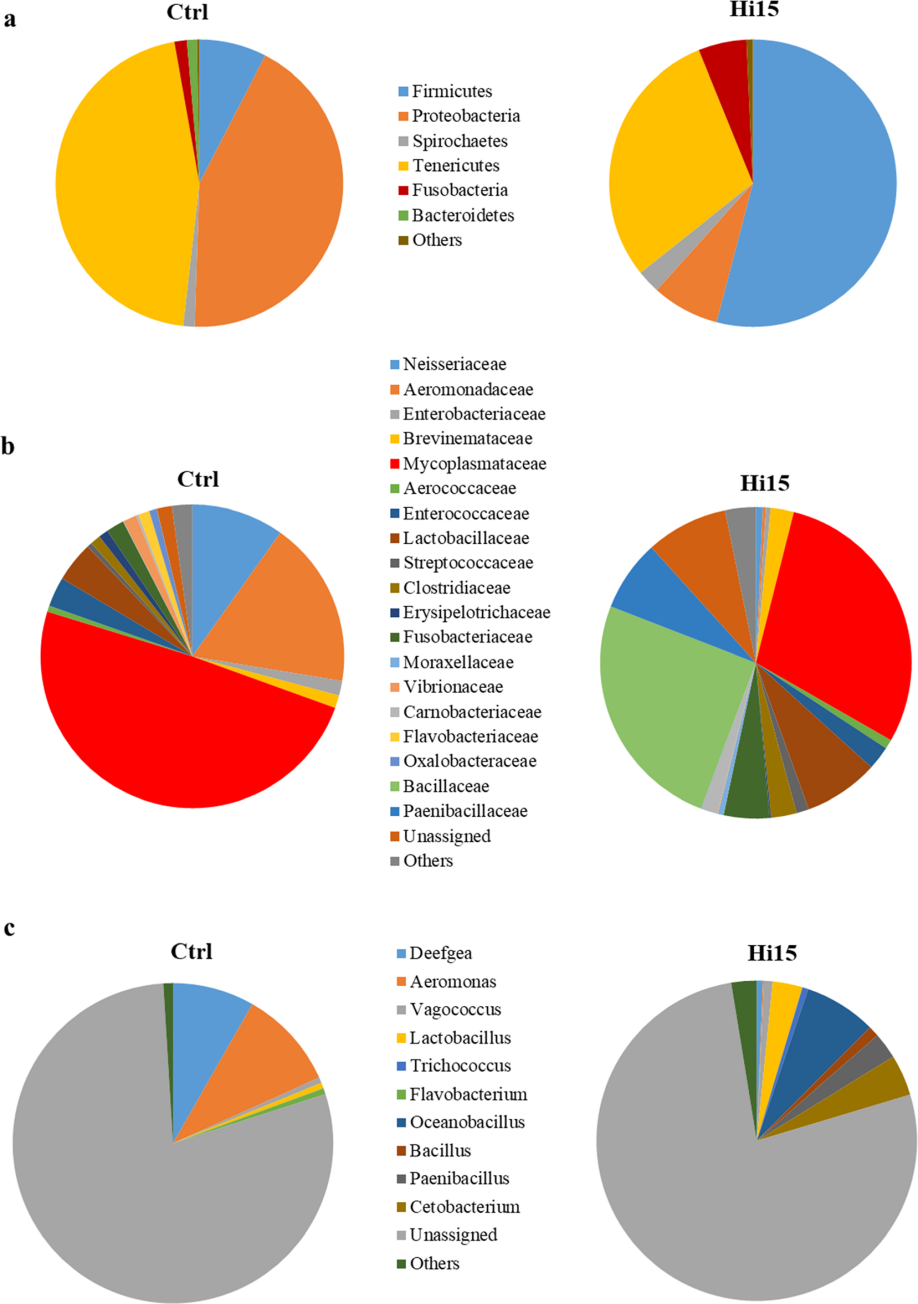
Relative abundance (%) of the most prevalent intestinal bacterial phyla (**a**), families (**b**), and genera (**c**) in each trout dietary group. In the figure, all taxa with an overall abundance of ≥ 0.5% were reported. Bacteria with lower abundance were pooled and indicated as “others”

Overall high-quality reads of 144,164 and 178,036 were taxonomically classified for Ctrl and Hi15 trout feeding groups, respectively. After removing the OTUs assigned to eukaryotic sequences, the most abundant bacterial taxa were mainly comprised of 6 phyla, 9 classes, 14 orders, 19 families, and 10 genera. The profiles of microbial communities at the phylum, family, and genus taxonomic levels for each trout group are shown in Fig. 2. The complete list of OTUs detected in intestinal samples is available as additional data in Online Resource 4.

The alpha rarefaction analysis of gut bacterial communities showed that indexes of species richness “Chao 1” and “Observed OTUs” were significantly higher in fish fed with Hi15 diet than in the control fish. Conversely, diet type did not affect either phylogenetic diversity (Faith PD) or entropy (Shannon and evenness) (Table 3).

**Table 3:** Alpha diversity metrics (rarefied at 10,780 sequences) of gut microbial communities of trout fed with Ctrl or Hi15 diets. All data are reported as mean values (*n* = 8) ± SD. Significant *p* values are in italic

Item	Ctrl	Hi15	*p* value
Observed OTUs	229.25 ± 57.68	370.50 ± 131.84	*0.02*
Chao 1	259.13 ± 70.03	421.93 ± 142.40	*0.01*
Faith-PD	4.52 ± 1.21	5.48 ± 1.77	0.11
Shannon	4.85 ± 0.42	5.39 ± 0.82	0.09
Evenness	0.62 ± 0.05	0.64 ± 0.05	0.60

**Fig. 3:**
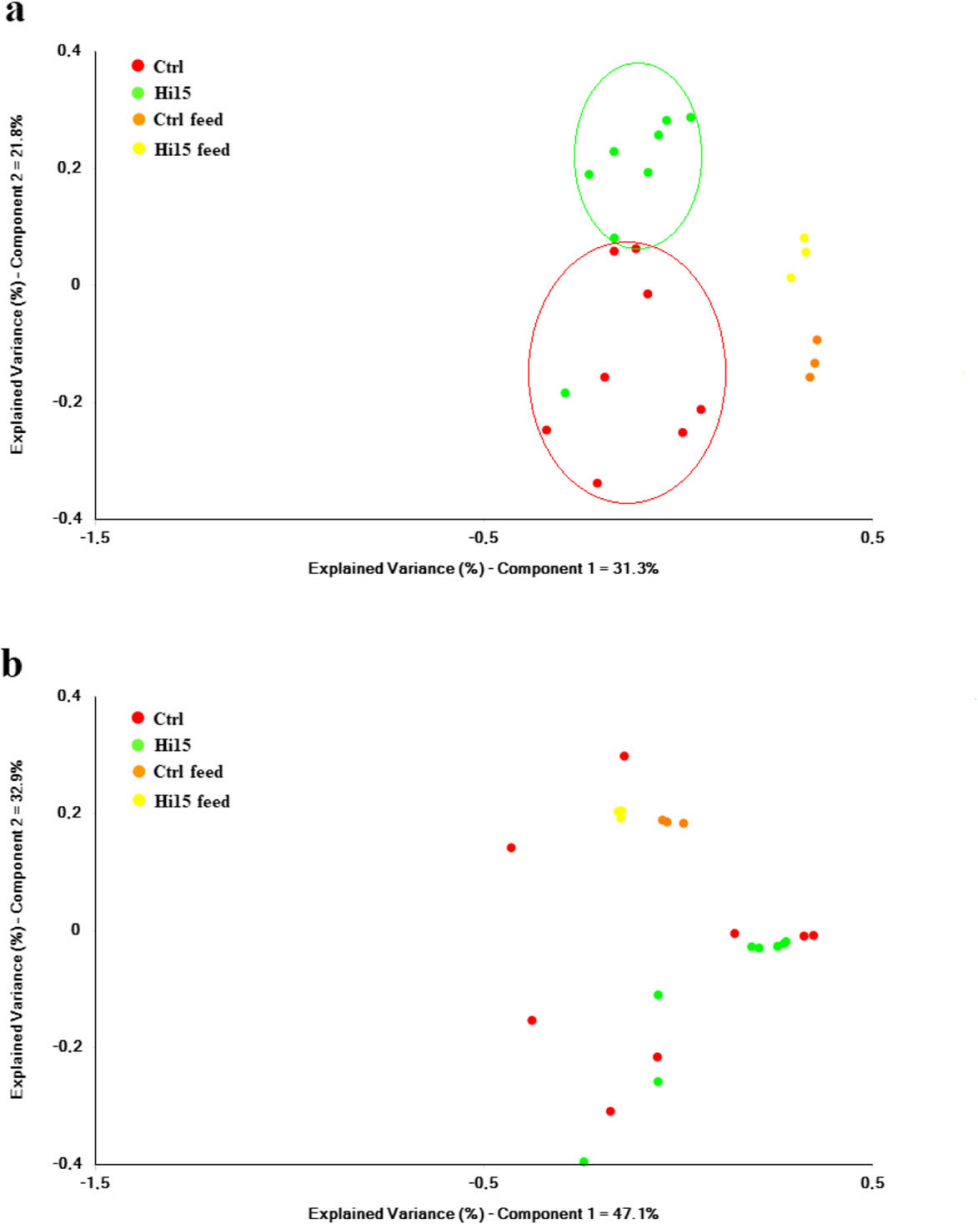
PCoA plot of unweighted (**a**) and weighted (**b**) UniFrac distances of gut microbial communities associated to two experimental dietary groups. Each dot represents an individual sample according to its microbial profile at genus level

Analysis of beta-diversity revealed an overall effect of diet on microbial communities in the presence/absence (unweighted UniFrac) (Fig. 3a), but not in relative abundance (weighted UniFrac), of specific OTUs (Fig. 3b). Principal coordinates analysis (PCoA) of unweighted UniFrac distances clearly showed that the intestinal microbiota of the Hi15 feeding group clustered separately from the Ctrl group; the two main components explain 53% of the observed variance (Fig. 3a). Additionally, intestinal communities were remarkably different from feed-associated bacterial ones, thus indicating that observed differences at the gut level were not simply a consequence of undigested feed that might have been present in the gastrointestinal tract. The PERMANOVA and ANOSIM tests confirmed the PCoA results, showing significant differences (*R* = 0.46, pseudo-*F* = 3.32, *q* < 0.05) in the composition of the microbiota between Ctrl and Hi15 feeding groups only in the unweight UniFrac analysis (Table 4). The relative abundances (%) of the most abundant taxa found in fish intestinal samples are reported in Table 5.

**Table 4:** ANOSIM and PERMANOVA test results for comparisons of gut microbiota composition between Ctrl and Hi15 feeding groups. Significant q-values (< 0.05) are shown in italic

Statistical test	Unweighted		Weighted	
**ANOSIM (permutation** ***N*** **= 999)**	***q*-value**	***R***	***q*-value**	***R***
Ctrl vs Hi15	*0.015*	0.42	0.247	0.06
Ctrl vs Ctrl diet	*0.042*	0.45	0.915	− 0.22
Hi15 vs Hi15 diet	*0.046*	0.46	0.174	0.47
Ctrl diet vs Hi15 diet	0.095	1.00	0.247	1.00
**PERMANOVA (permutation = 999)**	***q*-value**	**Pseudo-*F***	***q*-value**	**Pseudo-*F***
Ctrl vs Hi15	*0.009*	3.32	0.279	1.46
Ctrl vs Ctrl diet	*0.012*	4.87	0.346	1.22
Hi15 vs Hi15 diet	*0.009*	4.26	*0.036*	6.21
Ctrl diet vs Hi15 diet	0.119	9.02	0.228	58.18

**Table 5:** Mean relative abundance (%) ± SE (*n* = 8) of the most prevalent phyla, orders, classes, families, and genera found in the intestine of trout fed with two experimental diets. Significant *p* values (< 0.05) are shown in italic

	Ctrl	Hi15	*p* value
Phylum			
Firmicutes	7.58 ± 4.32	54.08 ± 14.58	*0.024*
Proteobacteria	42.95 ± 10.61	7.58 ± 1.42	*0.041*
Spirochaetes	1.29 ± 0.83	2.63 ± 1.64	0.563
Tenericutes	45.39 ± 11.44	29.56 ± 13.50	0.411
Fusobacteria	1.39 ± 0.68	5.42 ± 4.28	0.958
Bacteroidetes	1.14 ± 0.72	0.00 ± 0.00	
Class			
Clostridia	0.86 ± 0.63	3.59 ± 0.98	*0.031*
Alphaproteobacteria	10.77 ± 5.86	4.65 ± 1.25	0.793
Betaproteobacteria	10.92 ± 6.65	1.35 ± 0.92	0.103
Gammaproteobacteria	21.09 ± 11.45	1.59 ± 0.69	*0.018*
[Brevinematae]	1.29 ± 0.98	2.63 ± 1.84	0.563
Mollicutes	45.39 ± 11.56	29.55 ± 14.60	0.411
Bacilli	5.98 ± 3.53	50.35 ± 13.73	*0.024*
Fusobacteriia	1.39 ± 0.93	5.41 ± 4.82	0.958
Flavobacteriia	1.12 ± 0.93	0.00 ± 0.00	
Order			
Clostridiales	1.40 ± 1.10	3.81 ± 1.07	0.083
Neisseriales	9.84 ± 6.74	0.72 ± 0.56	0.178
Aeromonadales	17.76 ± 11.21	0.31 ± 0.20	*0.009*
Enterobacteriales	1.57 ± 0.98	0.49 ± 0.22	0.371
[Brevinematales]	1.36 ± 1.01	2.40 ± 1.57	0.636
Mycoplasmatales	49.22 ± 10.94	29.37 ± 14.71	0.320
Lactobacillales	9.27 ± 6.10	14.62 ± 4.60	0.339
Erysipelotrichales	1.02 ± 0.70	0.14 ± 0.06	0.220
Fusobacteriales	1.92 ± 1.35	4.74 ± 4.09	0.958
Vibrionales	1.44 ± 0.85	0.10 ± 0.07	0.215
Stramenopiles	0.77 ± 0.76	1.88 ± 1.88	0.543
Flavobacteriales	1.21 ± 1.01	0.00 ± 0.00	
Burkholderiales	1.13 ± 0.63	0.23 ± 0.08	0.956
Bacillales	0.02 ± 0.02	38.90 ± 11.18	*0.001*
Family			
Neisseriaceae	9.84 ± 6.74	0.72 ± 0.56	0.149
Aeromonadaceae	17.76 ± 11.21	0.31 ± 0.20	*0.009*
Enterobacteriaceae	1.57 ± 0.98	0.49 ± 0.22	0.371
Brevinemataceae	1.36 ± 1.01	2.40 ± 1.57	0.636
Mycoplasmataceae	49.23 ± 10.95	29.37 ± 14.71	0.173
Aerococcaceae	0.65 ± 0.58	0.97 ± 0.66	0.122
Enterococcaceae	3.16 ± 1.64	2.45 ± 0.80	0.902
Lactobacillaceae	4.30 ± 3.01	7.78 ± 2.48	0.226
Streptococcaceae	0.54 ± 0.49	1.30 ± 0.41	0.067
Clostridiaceae	1.09 ± 0.88	2.66 ± 0.68	0.083
Erysipelotrichaceae	1.02 ± 0.70	0.14 ± 0.06	0.220
Fusobacteriaceae	1.92 ± 1.35	4.74 ± 4.09	0.958
Moraxellaceae	0.05 ± 0.04	0.60 ± 0.52	0.717
Vibrionaceae	1.44 ± 0.85	0.08 ± 0.05	0.215
Carnobacteriaceae	0.26 ± 0.20	1.69 ± 0.95	*0.031*
Flavobacteriaceae	1.21 ± 1.01	0.00 ± 0.00	
Oxalobacteraceae	0.81 ± 0.46	0.04 ± 0.02	0.629
Bacillaceae	0.00 ± 0.00	25.17 ± 7.16	
Paenibacillaceae	0.00 ± 0.00	7.40 ± 2.25	
Genus			
*Deefgea*	8.26 ± 6.57	0.61 ± 0.56	0.178
*Aeromonas*	10.04 ± 6.66	0.11 ± 0.07	*0.007*
*Vagococcus*	0.56 ± 0.20	0.89 ± 0.35	0.439
*Lactobacillus*	0.55 ± 0.22	3.02 ± 1.03	*0.028*
*Trichococcus*	0.02 ± 0.02	0.60 ± 0.31	*0.018*
*Flavobacterium*	0.60 ± 0.43	0.00 ± 0.00	
*Oceanobacillus*	0.00 ± 0.00	7.27 ± 2.12	
*Bacillus*	0.00 ± 0.00	1.09 ± 0.28	
*Paenibacillus*	0.00 ± 0.00	2.65 ± 0.76	
*Cetobacterium*	0.00 ± 0.00	4.12 ± 4.11	

The gut microbial community of trout was dominated, regardless of the diet, by four phyla: Proteobacteria, Firmicutes, Tenericutes, and Fusobacteria (Fig. 3a). Of these, the amount of Firmicutes was positively influenced (*p* < 0.05) by dietary insect meal (Hi15 54%, Ctrl 7.6%) (Table 5). This was essentially due to the enrichment in bacteria belonging to the Clostridia (3.6%) and Bacilli (50%) class. On the contrary, the average relative abundance of Proteobacteria, mainly represented by Gammaproteobacteria, was significantly higher in Ctrl fish (43%) than in the Hi15 feeding group (7.6%). At order level, the only difference between two groups was in the amount of Aeromonadales and Bacillales (Table 5). The first taxon was more abundant in Ctrl samples, whereas Bacillales were enriched in fish fed Hi15 diet. Accordingly, Aeromonadaceae were particularly abundant in the gut of controls (18%), whereas Bacillaceae (25%) and Paenibacillaceae (7.4%) were solely found in trout receiving Hi15 diet (Fig. 3b, Table 5). The *Oceanobacillus*, *Bacillus*, *Paenibacillus*, and *Cetobacterium* genera were exclusive of the intestine of fish fed Hi meal. In the same dietary group, the amount of *Aeromonas* and *Lactobacillus* genera was significantly less and more abundant, respectively, in comparison to controls (Fig. 3c, Table 5).

### Prediction of metabolic pathways of gut bacterial communities

**Fig. 4:**
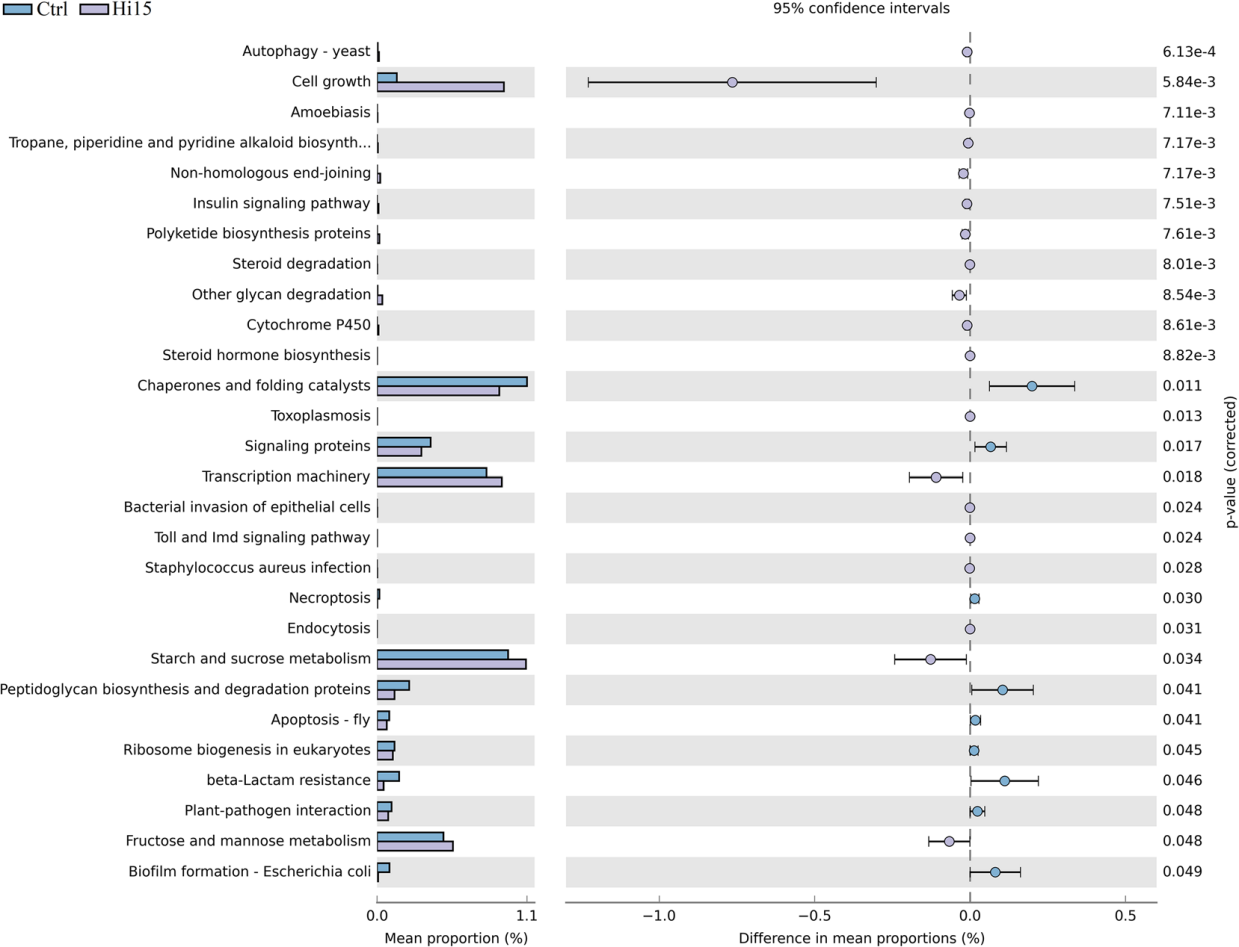
Predicted functional metagenomic pathways of trout gut microbiome, as identified by PICRUSt. The extended error bar graph and statistical analysis were made using STAMP bioinformatics software

PICRUSt was applied to predict the functional potential of the intestinal microbiome of rainbow trout. Level 3 KEGG orthologue function prediction was used. Our analysis revealed 217 predicted metabolic pathways (Online Resource 5). Among them, 28 were significantly different between the two dietary groups (Fig. 4). Metabolic inference from 16S rRNA gene sequencing data showed that dietary inclusion of Hi meal upregulated the abundance of genes responsible of pathways involved in starch and sugar metabolism and in the transcription processes. On the contrary, genes involved in the peptidoglycan biosynthesis and recycling and in the protein folding and biofilm formation were enhanced in the microbiome of control fish (Fig. 4).

## Discussion

The use of insect meal in fish feed is a way to respond to the problems of aquaculture industry related to the stability and reduction of feeding costs and to promote sustainable aquatic environment management. So far, several researches have shown that insect meal can partially replace fishmeal and completely replace soya bean meal that are commonly used in aquafeeds, without affecting fish growth performances, feed utilization, digestibility, and fillet quality (Magalhães et al. 2017; Renna et al. 2017; Bruni et al. 2018, 2020; Iaconisi et al. 2018; Terova et al. 2019). Indeed, the present research confirms what has been stated in previous studies on rainbow trout; i.e. defatted Hi meal is well accepted by trout and does not negatively affect fish growth and survival if it is included at levels up to 40% in the diet (Renna et al. 2017; Stadtlander et al. 2017; Bruni et al. 2018; Terova et al. 2019). Because fish are natural predators of insects, it is reasonable to assume that they are evolutionarily adapted for consuming them.

Nevertheless, fish growth performance is not the only outcome that defines a successful aquaculture practice; fish welfare has to be taken into account, too. In this prospect, intestinal microbiota, which directly affects the digestive functions and the immune response of the host should be considered a key indicator of a healthy fish (Ghanbari et al. 2015).

In line with our previous researches, the present study showed that Hi meal inclusion in the diet can modify fish gut microbiota, thus improving the health status of trout. In two recent studies in trout, we have reported that the partial substitution of dietary FM with 10%, 20%, or 30% of a defatted Hi meal had an important effect in modulating both the intestinal transient and resident bacterial communities (Rimoldi et al. 2019; Terova et al. 2019). As expected, the present metabarcoding analysis revealed that Firmicutes, Proteobacteria, and Tenericutes phyla were dominant in the gut of rainbow trout, regardless of the diet (Lyons et al. 2017a, b; Terova et al. 2019). The phylum Tenericutes is considered specifically adapted to the gastrointestinal environment of farmed rainbow trout. Several studies have reported that this phylum, with *Mycoplasma* being the dominant genus, is prominent in the distal intestine of rainbow trout as well as in other farmed salmonids (Lyons et al. 2017a; Huyben et al. 2018; Fogarty et al. 2019; Terova et al. 2019). Therefore, our data provide a further evidence of the importance of this genus in trout, thus corroborating the idea that this fish species could be a specific host for *Mycoplasma*.

Although gut bacterial communities were dominated by the same phyla irrespective of the diet, species richness (Chao 1 index, observed OTUs) was significantly increased by dietary supply of 15% of insect meal in our study. Previously, we found an increase of species richness only in the digesta-associated (allochthonous), but not in mucosa-associated (autochthonous), gut microbiota of rainbow trout fed with increasing levels of Hi meal (10–30%) (Rimoldi et al. 2019; Terova et al. 2019). Bruni et al. (2018) found instead, a higher species richness in autochthonous intestinal microbiota of trout fed a diet containing 20% of Hi meal. In any case, a higher microbial richness should be considered a positive effect, since it may potentially provide further metabolic capabilities to the host thus improving its health status (Borrelli et al. 2017).

Insect meals are rich in chitin, a form of insoluble fibre, which may act as prebiotic by selectively stimulating the growth of beneficial gut bacteria and promoting their colonization (Guerreiro et al. 2018). In the same way, biodiversity parameters were increased by dietary administration of krill or inclusion of 5–20% chitin in the diet of salmonids (Askarian et al. 2012; Ringø et al. 2012). Furthermore, chitin and its deacetylate derivate chitosan have antimicrobial properties and a bacteriostatic effect against several harmful Gram-negative bacteria (Nawaz et al. 2018).

Multivariate analysis of bacterial community’s diversity, based on unweighted UniFrac dissimilarity data, displayed a strong clustering of fish groups fed with Hi meal and with the control diet that were cleanly separated into uniformly distant regions. Our data confirm previous researches showing that the Hi meal inclusion in the diet causes a significant reduction of gut Proteobacteria, predominantly belonging to the Gammaproteobacteria class, in comparison to the control diet without insect meal (Huyben et al. 2019; Rimoldi et al. 2019; Terova et al. 2019). In particular, in line with those studies, our metagenomic analysis highlighted the dramatic shift from an high Proteobacteria to Firmicutes ratio in the gut of fish fed with the Ctrl diet to a low ratio in fish fed with the insect meal diet. The most dominant genus in the control fish gut was *Aeromonas*, which includes several Gram-negative bacteria commonly present in fresh water and potentially pathogenic for fish, as they can cause skin ulcerations. In the current study, intestinal abundance of *Aeromonas* in trout fed Hi15 was significantly reduced and this is in line with our findings on autochthonous intestinal microbiota of trout fed with Hi meal.

In another study of our group, microbiota of trout fed with Hi meal showed a reduction of Gammaproteobacteria, mainly represented by genera *Shewanella*, *Aeromonas*, *Citrobacter*, and *Kluyera* (Rimoldi et al. 2019). Similarly, Bruni et al. (2018) found a high abundance of OTUs related to the *Aeromonas* genus only in the control fish group, but not in the intestine of the insect-fed groups. An increase amount of *Aeromonas* genus with the Hi treatment has been recently reported only in Siberian sturgeon (*Acipenser baerii*) (Józefiak et al. 2019b).

We recorded an increase in the number of *Bacillus* and *Lactobacillus* genera in response to dietary insect meal. Proliferation of lactic acid bacteria (LAB) may be due to the prebiotic effect of chitin, and, as proposed by Bruni et al. (2018), it may indicate that chitin was a preferential growth substrate for LAB. Indeed, LAB play an important role in degrading fibres. Furthermore, they have an active role in host defence against pathogens, by producing bactericidal compounds, such as lactic acid, hydrogen peroxide, bacteriocins, and biosurfactants, which prevent pathogen colonization of the intestinal epithelial surface (Ringø and Gatesoupe 1998; Corr et al. 2007; Gudiña et al. 2015; Ringø et al. 2018). Even the increased amount of *Bacillus* represents a positive effect of dietary chitin deriving from insect meal. Chitin, indeed, may have increased the proliferation of chitinolytic bacteria, since several *Bacillus* species have been shown to secrete chitinase (Cody 1989). Together with LAB, the *Bacillus* genus is one of the most common probiotics used in aquaculture to enhance host immune response and disease resistance. Up to date, several studies have demonstrated the immunomodulatory effects of *Bacillus subtilis* in fish (Salinas et al. 2005; Newaj-Fyzul et al. 2007; Cerezuela et al. 2013) and there are several evidences documenting that the use of insect meals from *H. illucens* may positively modulate trout gut microbiota, increasing LAB and Bacilli amount in both mucosa- and digesta-associated microbiota (Bruni et al. 2018; Huyben et al. 2019; Terova et al. 2019; Józefiak et al. 2019a).

In addition to taxonomic characterization of gut microbiota in response to dietary insect meal, this study investigated the functional potential of the intestinal microbiome of rainbow trout using the computational approach PICRUSt (Langille et al. 2013). Indeed, the use of dietary insect meal clearly affected the structure of trout intestine–associated microbial community (what’s there?) but, to understand the intrinsic processes that lead to similar functionality, it is necessary to search the connections between individual microbiota (what are they doing?) and the corresponding metabolic phenotype (Piazzon et al. 2017).

Gut microbes carry out a multitude of biochemical reactions, which play a critical role in host nutrition by contributing to the digestion of several dietary ingredients. In agreement with Lyons et al. (2017a), we found that the principal functional pathways associated with bacterial communities of trout intestine, regardless of the diet, were metabolism, cellular processes, membrane transport, and genetic information processing.

However, based on metagenome prediction, trout fed with insect meal showed an enhancement of pathways involved in sugar and starch metabolism. Members of the phylum Firmicutes are known to play a pivotal role in the fermentation of dietary carbohydrates (Corrigan et al. 2015). In our case, the increase of sugar metabolism observed in the Hi group of trout could be reasonably correlated to the higher presence of Bacilli that typify the intestinal microbiota of these fish. The fermentation of dietary carbohydrates and resistant starches by the intestinal microbiota leads to the formation of a variety of beneficial substances, including short-chain fatty acids (SCFAs). It is well established that SCFAs (mainly acetate, propionate, and butyrate), in addition to being energy sources for colonocytes, promote fish intestinal health (Hamer et al. 2008; Koh et al. 2016; Rimoldi et al. 2016). Furthermore, the increased ability of gut microbiome to utilize dietary carbohydrates could be an interesting approach to improve feed digestibility in trout that is known as a poor user of dietary carbohydrates and fibres (Wilson 1994; Polakof et al. 2012). In fact, *Bacillus* genera are widely used as probiotics in aquaculture to increase feed absorption and digestion (Soltani et al. 2019).

On the contrary, intestinal microbiome of trout fed with the Ctrl diet showed an increased capacity for peptidoglycan synthesis. Peptidoglycan is the major structural component of the cell wall of both Gram-positive and Gram-negative bacteria. It is the major wall structural component of the most pathogenic bacteria and it is considered a proinflammatory molecule that stimulates host innate immune response (Mogensen 2009). In human, for instance, functional analysis of the faecal microbiome of healthy individuals and atherosclerosis patients revealed an increase in the peptidoglycan synthesis gene in the afflicted population (Karlsson et al. 2012). It means that the increased capacity for peptidoglycan synthesis might contribute to the chronic inflammation of the atherosclerotic arterial walls.

The hypothesis that control fish in the present study were affected by an inflammatory status seems to be supported by the increase of gene pathways of chaperones and protein-folding catalysts found in their intestinal microbiota. Indeed, secretion of chaperones and protein-folding catalysts (foldase) from prokaryote cells acts as intercellular signal, principally for leukocytes. Chaperones and foldase have been defined “moonlighting” proteins since they may act as homeostatic immune regulators and, under certain circumstances, contribute to tissue pathology as well (Henderson and Pockley 2010).

Effectively, Proteobacteria dominated intestinal microbiome of control trout, whereas Firmicutes were scarcely represented. This phylum was mainly represented by Gammaproteobacteria class, which includes important disease-causing pathogens of fish. Among these, *Aeromonas* resulted particularly abundant in the intestine of fish fed with Ctrl diet, possibly as a sign of intestinal dysbiosis or disease.

In summary, the present research reinforces the insights of previous studies conducted by us and other groups showing that insect proteins can have beneficial effects on intestinal microbiota of fish. The inclusion of 15% of *H. illucens* led to an increase in the total number of Firmicutes, mainly represented by Bacilli class, and to a drastic reduction of Proteobacteria. Beneficial genera, such as *Lactobacillus* and *Bacillus*, were enriched in the gut of fish fed with an insect-based diet, while the number of bacteria assigned to the pathogenic *Aeromonas* genus was drastically reduced in the same fish group. The metagenome functional data provided evidence that dietary IM inclusion can shape the metabolic activity of trout gut microbiota. In particular, intestinal microbiome of trout fed with insect meal may have the capacity to complement the endogenous digestive enzymes, thus improving dietary carbohydrates utilization. Therefore, *H. illucens* meal is a promising alternative protein source for trout nutrition, able to modulate gut microbial community by increasing the abundance of some bacteria taxa that are likely to play a key role in fish health.

## Supplementary information

ESM 1 (PDF 213 kb)

ESM 2 (PDF 37 kb)

ESM 3 (PDF 37 kb)

ESM 4 (PDF 156 kb)

ESM 5 (PDF 232 kb)

## Data Availability

All sequencing data were submitted to the European Nucleotide Archive (EBI ENA) public database, under the accession code PRJEB38953.
