# Supplementary material for: Intestinal microbial communities of rainbow trout (Oncorhynchus mykiss) may be improved by feeding a Hermetia illucens meal/low-fishmeal diet
Source: Fish Physiol Biochem. 2021 Jan 3;47(2):365–80. doi: 10.1007/s10695-020-00918-1 (PMC8026480; doi:10.1007/s10695-020-00918-1)
Supplement: Supplementary file 1 — (PDF 213 kb) [file 10695_2020_918_MOESM1_ESM.pdf]

Fish Physiology and Biochemistry.

Taxonomic and functional characterization of intestinal microbial communities of rainbow trout (*Oncorhynchus mykiss*) fed with *Hermetia illucens* meal as alternative protein source. Simona Rimoldi, Micaela Antonini, Laura Gasco, Federico Moroni, and Genciana Terova. Department of Biotechnology and Life Sciences, University of Insubria, Via J.H. Dunant, 3, 21100 Varese, Italy. genciana.terova@uninsubria.it

Supplementary Fig. 1 Rarefaction curves of chao1 alpha diversity index.

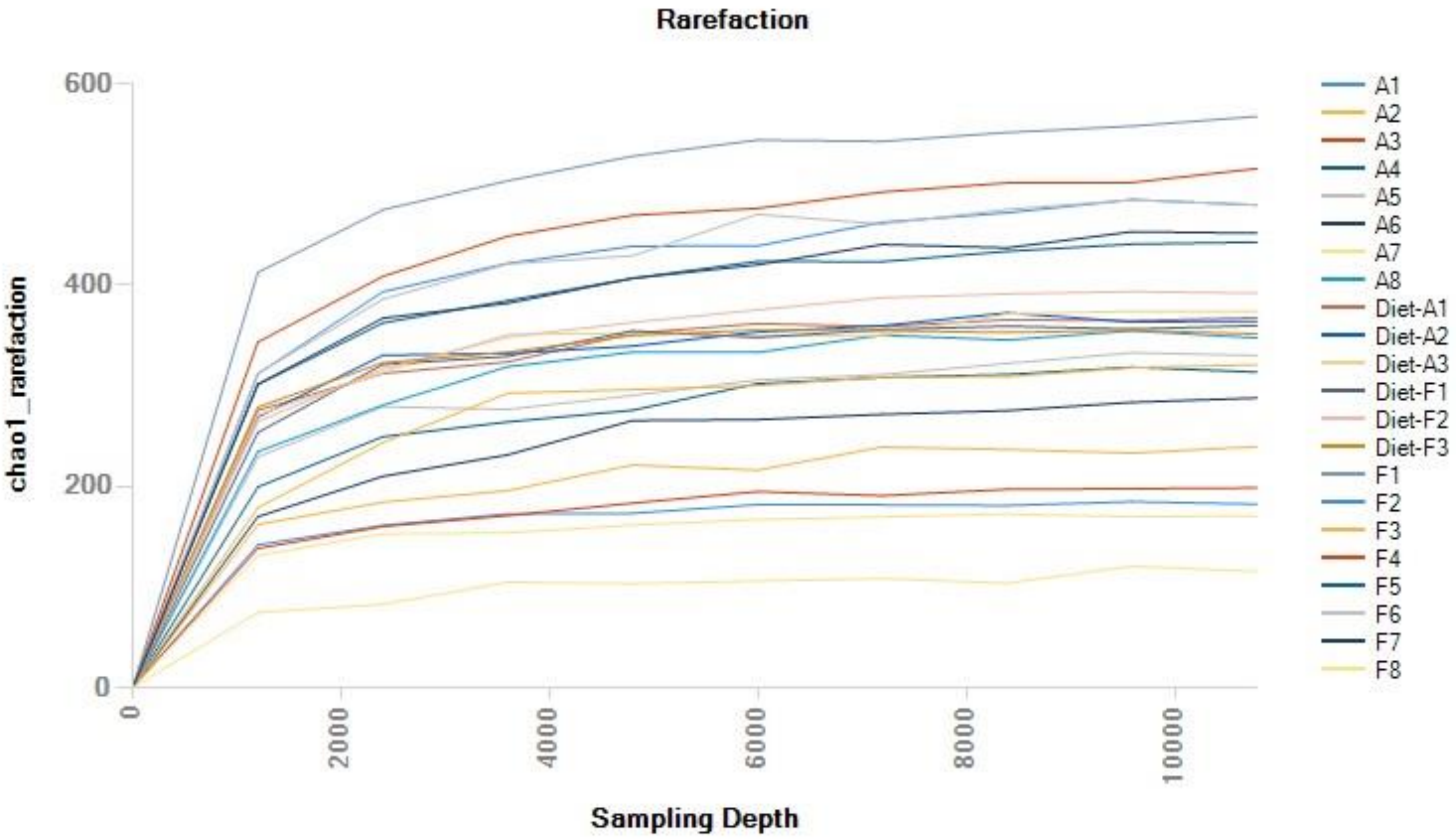

Rarefaction
chao1_rarefaction
600
400
200
0
0
2000
4000
6000
8000
10000
Sampling Depth
A1
A2
A3
A4
A5
A6
A7
A8
Diet-A1
Diet-A2
Diet-A3
Diet-F1
Diet-F2
Diet-F3
F1
F2
F3
F4
F5
F6
F7
F8
