# Supplementary material for: Intestinal microbial communities of rainbow trout (Oncorhynchus mykiss) may be improved by feeding a Hermetia illucens meal/low-fishmeal diet
Source: Fish Physiol Biochem. 2021 Jan 3;47(2):365–80. doi: 10.1007/s10695-020-00918-1 (PMC8026480; doi:10.1007/s10695-020-00918-1)
Supplement: Supplementary file 2 — (PDF 37 kb) [file 10695_2020_918_MOESM2_ESM.pdf]

Fish Physiology and Biochemistry

Taxonomic and functional characterization of intestinal microbial communities of rainbow trout (*Oncorhynchus mykiss*) fed with *Hermetia illucens* meal as alternative protein source.

Simona Rimoldi, Micaela Antonini, Laura Gasco, Federico Moroni, and Genciana Terova. Department of Biotechnology and Life Sciences, University of Insubria, Via J.H. Dunant, 3, 21100 Varese, Italy.
genciana.terova@uninsubria.it

Supplementary data file 1: List of OTUs found in feed samples. The relative abundance (%) of each OTU has been reported.

OTUs	Ctrl feed	Ctrl feed	Ctrl feed	Hi15 feed	Hi15 feed	Hi15 feed
Unknown Stramenopiles	0,26024	0,27357	0,33721	0,09192	0,10222	0,09463
Unknown Clostridiales	0,00139	0,00192	0,00116	0,00215	0,00206	0,00254
Unknown mitochondria	0,06098	0,06009	0,06667	0,04359	0,05317	0,04421
Aeromonas	0,00352	0,00274	0,00305	0,00017	0,0006	0,00035
Unknown Enterobacteriaceae	0,00176	0,00141	0,00087	0,00112	0,00052	0,00079
Unknown Mycoplasmataceae	0	0	0	0	0,00017	0
Facklamia	0,00213	0,00178	0,0016	0,00319	0,00318	0,00429
Vagococcus	0,17646	0,15532	0,13116	0,29661	0,28769	0,30237
Lactobacillus	0,09889	0,1107	0,09667	0,06633	0,06761	0,07993
Weissella	0,0089	0,00888	0,0085	0,00715	0,00644	0,00595
Streptococcus	0,00565	0,0054	0,00494	0,005	0,00481	0,00481
Unknown Clostridiaceae	0,00158	0,00155	0,0016	0,00215	0,00215	0,00184
Unknown Peptostreptococcaceae	0,00009	0,00044	0,00044	0	0,00017	0,00018
Erysipelothrix	0,00445	0,00385	0,00356	0,01223	0,01194	0,01497
Unknown Fusobacteriaceae	0,00259	0,00511	0,00516	0,00026	0,00052	0,00053
Unknown Aeromonadaceae	0	0,00074	0	0	0,00026	0
Pantoea	0	0	0,00015	0,00026	0	0
Photobacterium	0	0,00022	0	0	0	0
Trichococcus	0,00009	0	0	0	0,00026	0
Shewanella	0,01297	0,01125	0,00821	0,00637	0,00533	0,0056
Clostridium	0,00046	0,00081	0,00094	0,00103	0,00155	0,0014
Unknown [Tissierellaceae]	0,00139	0,00096	0,00073	0,00172	0,00198	0,00184
Unknown Erysipelotrichaceae	0,00547	0,00659	0,00458	0,00844	0,0085	0,00963
Vibrio	0,33735	0,33343	0,31208	0,42746	0,415	0,40165
Oceanobacillus	0	0	0	0,00525	0,0049	0,00455
Halomonas	0,00222	0,0017	0,0016	0,00052	0,00069	0,00018
Acinetobacter	0,00408	0,00392	0,00334	0,00086	0,00094	0,00131
Corynebacterium	0	0	0	0,00017	0	0
Unknown Lachnospiraceae	0	0	0	0,00086	0,00026	0,00053
Shigella	0	0,00052	0,00036	0,00017	0	0,00044
Morganella	0,00102	0,00074	0,00044	0,00034	0,00069	0,00088
Myroides	0,0025	0,00237	0,00138	0	0,00043	0
Megasphaera	0,00046	0,0003	0,00029	0,00034	0,00034	0,00053
Proteus	0,00185	0,00222	0,00196	0,00103	0,00198	0,00175
Providencia	0,00009	0,00007	0,00015	0,00017	0,00017	0,00026
Pasteurella	0,00037	0,00044	0,00044	0,00052	0,00069	0,00044
Wohlfahrtiimonas	0,00102	0,00089	0,0008	0,0006	0,00146	0,00096
Bifidobacterium	0	0,00007	0	0,00034	0	0
Unknown Actinomycetales	0	0	0	0,00164	0,00206	0,00123
Actinomyces	0	0	0	0,00017	0,00017	0,00018
Unknown Beutenbergiaceae	0	0	0	0,00155	0,00198	0,00149
Unknown Bacteroidales	0	0	0	0,00017	0,00043	0,00035
Parabacteroides	0	0	0	0,00043	0,00026	0,00053
Unknown Bacillales	0	0	0	0,00121	0,00103	0,00201
Unknown Bacillaceae	0	0	0	0,00078	0,00146	0,00061
Bacillus	0	0	0	0,00112	0,0006	0,0007
Virgibacillus	0	0	0	0,00069	0,00026	0
Paenibacillus	0	0	0	0,00379	0,00258	0,00333

Enterococcus	0	0	0	0,00009	0	0,00026
Gracilibacillus	0	0	0	0	0,00017	0
Staphylococcus	0	0	0	0	0,00017	0
Unknown Thermoactinomycetaceae	0	0	0	0	0,00017	0
