# Supplementary material for: Intestinal microbial communities of rainbow trout (Oncorhynchus mykiss) may be improved by feeding a Hermetia illucens meal/low-fishmeal diet
Source: Fish Physiol Biochem. 2021 Jan 3;47(2):365–80. doi: 10.1007/s10695-020-00918-1 (PMC8026480; doi:10.1007/s10695-020-00918-1)
Supplement: Supplementary file 3 — (PDF 37 kb) [file 10695_2020_918_MOESM3_ESM.pdf]

Fish Physiology and Biochemistry

Taxonomic and functional characterization of intestinal microbial communities of rainbow trout (*Oncorhynchus mykiss*) fed with *Hermetia illucens* meal as alternative protein source. Simona Rimoldi, Micaela Antonini, Laura Gasco, Federico Moroni, and Genciana Terova. Department of Biotechnology and Life Sciences, University of Insubria, Via J.H. Dunant, 3, 21100 Varese, Italy. genciana.terova@uninsubria.it

Supplementary Table 1. Mean relative abundance (%) ± SE (n = 3) of the most prevalent phyla, orders, classes, families, and genera found in feed samples. Significant *p*-value (< 0.05) are in bold.

**Phylum**	**Ctrl feed**			**Hi15 feed**			***p*-value**
Firmicutes	40.43	±	0.90	46.94	±	0.97	**0.008**
Proteobacteria	58.66	±	0.85	52.51	±	0.92	**0.008**
**Class**							
Alphaproteobacteria	8.93	±	0.61	5.25	±	0.36	**0.005**
Gammaproteobacteria	50.20	±	0.26	47.76	±	0.78	**0.041**
Bacilli	38.62	±	0.86	43.99	±	0.83	**0.011**
Erysipelotrichi	1.34	±	0.06	2.45	±	0.15	**0.002**
**Order**							
Lactobacillales	42.04	±	0.68	44.58	±	0.80	0.072
Erysipelotrichales	1.46	±	0.06	2.56	±	0.15	**0.002**
Pseudomonadales	0.58	±	0.01	0.12	±	0.02	0.081
Vibrionales	50.71	±	0.83	48.41	±	0.89	0.131
Alteromonadales	1.66	±	0.15	0.67	±	0.03	**0.002**
Bacillales	0.00	±	0.00	1.38	±	0.06	
**Family**							
Aeromonadaceae	0.52	±	0.00	0.05	±	0.02	0.081
Enterobacteriaceae	0.70	±	0.03	0.41	±	0.03	**0.003**
Enterococcaceae	23.77	±	1.18	34.53	±	0.32	**0.001**
Lactobacillaceae	15.80	±	0.63	8.32	±	0.49	**0.001**
Leuconostocaceae	1.36	±	0.04	0.76	±	0.04	**0.000**
Streptococcaceae	0.82	±	0.01	0.57	±	0.01	0.091
Erysipelotrichaceae	1.46	±	0.06	2.56	±	0.15	**0.002**
Fusobacteriaceae	0.67	±	0.15	0.05	±	0.01	**0.004**
Moraxellaceae	0.58	±	0.01	0.12	±	0.02	**0.000**
Vibrionaceae	50.71	±	0.83	48.43	±	0.90	0.136
Shewanellaceae	1.66	±	0.15	0.67	±	0.03	**0.002**
Bacillaceae	0.00	±	0.00	0.82	±	0.07	
**Genus**							
Vagococcus	16.46	±	1.37	31.01	±	0.36	**0.001**
Lactobacillus	10.89	±	0.45	7.48	±	0.45	**0.006**
Weissella	0.93	±	0.01	0.68	±	0.04	**0.003**
Streptococcus	0.57	±	0.02	0.51	±	0.01	0.058
Erysipelothrix	0.42	±	0.03	1.37	±	0.10	**0.000**
Shewanella	1.15	±	0.15	0.60	±	0.03	**0.016**
Vibrio	34.95	±	0.77	43.52	±	0.79	**0.016**
Oceanobacillus	0.00	±	0.00	0.51	±	0.02	
