# Supplementary material for: Intestinal microbial communities of rainbow trout (Oncorhynchus mykiss) may be improved by feeding a Hermetia illucens meal/low-fishmeal diet
Source: Fish Physiol Biochem. 2021 Jan 3;47(2):365–80. doi: 10.1007/s10695-020-00918-1 (PMC8026480; doi:10.1007/s10695-020-00918-1)
Supplement: Supplementary file 4 — (PDF 156 kb) [file 10695_2020_918_MOESM4_ESM.pdf]

Fish Physiology and Biochemistry

Taxonomic and functional characterization of intestinal microbial communities of rainbow trout (*Oncorhynchus mykiss*) fed with *Hermetia illucens* meal as alternative protein source.

Simona Rimoldi, Micaela Antonini, Laura Gasco, Federico Moroni, and Genciana Terova. Department of Biotechnology and Life Sciences, University of Insubria, Via J.H. Dunant, 3, 21100 Varese, Italy. genciana.terova@uninsubria.it

Supplementary data file 2 : List of OTUs found in intestinal samples of trout fed with experimental diets. The relative abundance (%) of each OTU has been reported.

**OTUs**	Ctrl	Ctrl	Ctrl	Ctrl	Ctrl	Ctrl	Ctrl	Ctrl	Hi15	Hi15	Hi15	Hi15	Hi15	Hi15	Hi15	Hi15
Unknown Stramenopiles	0,051	0,0918	0,2321	0,2915	0,8296	0,8804	0,0083	0,5565	0,51986	0,6749	0,2828	0,5501	0,67065	0,1681	0,68903	0,00218
Unknown Clostridiales	9E-05	0	0	0	0	0,0011	0	0,0003	0,00893	0,0051	0,0008	0,00289	0,0001	0,0008	0,00412	0
Unknown mitochondria	0,0014	0,0032	0,0061	0,0196	0,0523	0,0507	0,00011	0,0359	0,01862	0,0278	0,0139	0,01964	0,01363	0,0111	0,03232	0
Unknown Betaproteobacteria	0,0089	0	0	0,0001	0,0016	0,0007	0,01075	0,002	0	0	0	0	0	0,0193	0,00036	0
Deefgea	0,5251	0,0001	0	0,0031	0,0132	0	0,10784	0,0023	0,00071	0,0007	0	0	0,00174	0,0442	0	0
Aeromonas	0,0132	0,0009	0,5467	0,0106	0,0008	0,0008	0,13133	0,0905	0,00555	0	0	0,00221	0	0	0,00054	0
Unknown Enterobacteriaceae	0,0645	0,0013	0,0017	0	0,0002	0,0005	0,00967	0,0044	0,00235	0,0007	0	0,00016	0,00085	0,0001	0,00036	0
Serratia	0,0137	0	0	0	0	0	0	0	0	0	0	0	0	0	0	0
Unknown Brevinemataceae	0,0018	0,0087	0,0002	0,0576	0,0011	9E-05	0,00085	0,0009	0,00147	0	0,0078	0	0,00124	0,1256	0,01019	0,00719
Unknown Mycoplasmataceae	0,3203	0,7414	0,0077	0,5698	0,0472	0,02	0,6842	0,1769	0,0275	0,0123	0,6476	0,0052	0,01995	0,2412	0,00299	0,98905
Actinotelluria	0	0,0006	0	0	0	0	0	0	0	0	0	0	0	0	0	0
Unknown Cyanobacteria	0	0,0007	0	0	0	0	0	0	0	0	0	0	0	0,0029	0	0
Facklamia	0	0,0003	0	0	0,0005	0,0032	0	0,0002	0,00284	0,0011	0,0008	0,00347	0,01732	0,0002	0,00041	0
Vagococcus	0	0,0015	0,001	0,0084	0,0093	0,0086	0	0,0141	0,02923	0,0065	0,0029	0,01507	0,00722	0,0016	0,00638	0
Lactobacillus	0	0,0056	0,0007	0,0028	0,0081	0,017	0	0,0073	0,08824	0,0425	0,0062	0,03356	0,03384	0,0068	0,02368	0
Weissella	0	0,0003	0	0,0004	0,0002	0,0002	0	0,0006	0,00218	0,003	0,0001	0,00263	0	0	0,00036	0
Streptococcus	0	0,0007	0	0	0,0004	0,0027	0	0	0,00857	0,0092	0,0009	0,00536	0,00796	0,0016	0,00376	0
Turicibacter	0	0,0003	0	0	0	9E-05	0	0	0,00298	0,0004	0,0008	0,00011	0,0004	0,0009	0,00045	0
Unknown Clostridiaceae	0	0,0011	0,0005	0	0,0009	0,0025	0	0,0001	0,01368	0,0086	0,0026	0,00898	0,00751	0,0041	0,00761	0,00007
Unknown Peptostreptococcaceae	0	0,0001	0	0	0,0004	0	0	0,0003	0,00173	0,0011	0,0004	0,00105	0,0005	0,0006	0,00086	0
Erysipelothrix	0	0,0008	0,0017	0,0013	0,0069	0,0007	0	0,003	0	0	0,0014	0,00147	0,00075	0	0,00027	0,0001
Unknown Fusobacteriaceae	0	0,001	0,0003	0,004	0,0132	0,0009	0	0,0085	0,00204	0,0111	0	0,00084	0	0	0,00081	0
Unknown Isosphaeraceae	0	0,0161	0	0	0	0	0	0	0	0	0	0	0	9E-05	0	0

Mycoplana	0	0,0007	0	0	0	0	0	0	0	0	0	0	0	0	0	0
Unknown Rhizobiales	0	0,0009	0	0	0	0	0	0	0	0	0	0	0	0,0022	0	0
Methylobacterium	0	0,0007	0	0	0	0	0	0	0	0	0	0	0	0	0	0
Unknown Rhodobacteraceae	0	0,0006	0	0	0	0	0	0	0	0	0	0	0	0	0	0
Paracoccus	0	0,0003	0	0	0	0	0	0	0,00036	0	0	0,00058	0	0	0	0
Roseomonas	0	0,0007	0	0	0	0	0	0	0	0	0	0	0	0	0	0
Phytophthora	0	0,0004	0	0	0	0	0	0	0,00542	0	0	0	0	0	0	0
Unknown Aeromonadaceae	0	6E-05	0,201	0	0,0002	5E-05	0,03822	0,0223	0,0016	0	0	0,00084	0	0	0	0
Pantoea	0	0,0003	0	0	0	0	0	0	0	0	0	0,00011	0	0	0,00045	0
Enhydrobacter	0	0,0001	0	0	0,0002	0	0	0	0	0	0	0,00089	0,01329	0	0	0
Pseudomonas	0	0,0006	0	0,0067	0	0	0	0,0043	0	0	0	0,00074	0	0	0	0
Photobacterium	0	0,0002	0,0001	0,0004	0,0025	0,0008	0	0,0004	0	0,0011	0,0002	0	0	0	0,00045	0
Stenotrophomonas	0	0,0002	0	0	0	0	0	0	0	0	0	0,00068	0	0	0	0
Mycoplasma	0	0,1177	0	0	0	0	0	0	0	0	0	0	0	0	0	0
Trichococcus	0	0	0,0001	0	0,0005	0,0011	0	0	0,00933	0,0053	0,0005	0,00341	0,02543	0,0005	0,00208	0
Shewanella	0	0	0,0003	0,0007	0,0017	9E-05	0	0,0006	0,00067	0,0005	0	0,00021	0	0	0,00027	0
Flavobacterium	0	0	0	0,0039	0	0	0,00859	0,0335	0	0	0	0	0	0	0	0
Clostridium	0	0	0	0,0002	0,0007	0,0024	0,00011	0	0,01066	0,0017	0,0012	0,00788	0,0003	0,0073	0,00444	0,00007
Unknown [Tissierellaceae]	0	0	0	0,0001	0	0	0	0,0004	0	0	0	0	0	0	0,00054	0
Unknown Erysipelotrichaceae	0	0	0	0,0004	0	0	0	0	0	0	0	0,00079	0	0	0,00009	0
Sutterella	0	0	0	0,0002	0	0	0	0	0,00067	0	0	0	0	0	0	0
Unknown Comamonadaceae	0	0	0	0,0007	0	0	0	0,0034	0,00031	0	0	0	0	0	0	0
Unknown Oxalobacteraceae	0	0	0	0,0099	0	0,0009	0	0,0113	0	0,0003	0	0	0,00035	0	0	0
Herminiimonas	0	0	0	0,0007	0	0	0	0,0003	0	0	0	0	0	0	0	0
Arcobacter	0	0	0	0,0006	0	0	0	0,001	0	0	0	0	0	0	0	0
Vibrio	0	0	0	0,0063	0,0058	9E-05	0	0,0086	0	0	0	0,00011	0	0	0,00009	0
Oceanobacillus	0	0	0	0	0,0002	0	0	0	0,11836	0,0855	0,0158	0,16742	0,06658	0,0137	0,0977	0,00086
Unknown Caulobacteraceae	0	0	0	0	5E-05	0	0	0	0,00053	0	0	0	0	0	0	0
Reyranella	0	0	0	0	0,0001	0	0	0	0	0,0004	0	0	0	0,0002	0	0
Rubrivivax	0	0	0	0	0,0001	0,0006	0	0	0,00031	0,0006	0	0,0011	0,00159	0	0,00054	0
Unknown Desulfovibrionaceae	0	0	0	0	0,0017	0	0	0	0	0	0	0	0	0	0	0
Halomonas	0	0	0	0	0,0002	0	0	0	0,00036	0	0	0	0	0	0	0

Acinetobacter	0	0	0	0	0,0002	0	0	0,0001	0,00084	0	0	0,00068	0	0	0	0
Corynebacterium	0	0	0	0	0	0,0003	0	0	0,00138	0	0	0	0,00095	0,0002	0,00054	0
Unknown Lactobacillales	0	0	0	0	0	0,0015	0	0	0,00102	0,0014	0,0002	0,00105	0,0002	0	0,00063	0
Unknown Lachnospiraceae	0	0	0	0	0	0,0001	0	0	0	0,0006	0	0,00016	0	0	0	0
Agrobacterium	0	0	0	0	0	0,0005	0	0	0	0	0	0	0	0	0	0
Acidovorax	0	0	0	0	0	0,0001	0	0	0	0,0004	0	0	0	0	0	0
Shigella	0	0	0	0	0	0,0007	0	0	0,00475	0,0011	0	0	0,00219	0,0002	0,00023	0
Haemophilus	0	0	0	0	0	0,0006	0	0	0	0	0	0	0	0	0	0
Flectobacillus	0	0	0	0	0	0	0	0,0008	0	0	0	0	0	0	0	0
Rhodoferax	0	0	0	0	0	0	0	0,0007	0	0	0	0	0	0	0	0
Sphaerotilus	0	0	0	0	0	0	0	0,0011	0	0	0	0	0	0	0	0
Janthinobacterium	0	0	0	0	0	0	0	0,003	0	0	0	0	0	0	0	0
Unknown Methylophilales	0	0	0	0	0	0	0	0,001	0	0	0	0	0	0	0	0
Unknown Methylophilaceae	0	0	0	0	0	0	0	0,0025	0	0	0	0	0	0	0	0
Unknown Neisseriaceae	0	0	0	0	0	0	0	0,0007	0,00133	0	0	0	0	0	0	0
Morganella	0	0	0	0	0	0	0	0,0001	0	0	0	0	0,00114	0	0	0
Bifidobacterium	0	0	0	0	0	0	0	0	0	0,0002	0	0	0	0	0	0
Unknown Actinomycetales	0	0	0	0	0	0	0	0	0	0,0003	0	0,00042	0,00214	0	0	0
Actinomyces	0	0	0	0	0	0	0	0	0,00013	0	0	0,00016	0	0	0	0
Unknown Beutenbergiaceae	0	0	0	0	0	0	0	0	0	0	0	0,001	0	0	0,00023	0
Unknown Bacteroidales	0	0	0	0	0	0	0	0	0,00022	0	0	0	0	0	0	0
Parabacteroides	0	0	0	0	0	0	0	0	0	0	0	0	0	0	0	0
Unknown Bacillales	0	0	0	0	0	0	0	0	0,03128	0,0229	0,004	0,04674	0,02349	0,0047	0,03069	0,00014
Unknown Bacillaceae	0	0	0	0	0	0	0	0	0,00973	0,0096	0,0018	0,01612	0,01592	0,0015	0,01091	0,00014
Bacillus	0	0	0	0	0	0	0	0	0,01435	0,0142	0,0016	0,02053	0,01587	0,003	0,01517	0
Virgibacillus	0	0	0	0	0	0	0	0	0,00502	0,0032	0,001	0,00231	0,00139	0,0003	0,00186	0
Paenibacillus	0	0	0	0	0	0	0	0	0,03199	0,0376	0,0045	0,06039	0,02448	0,0069	0,0402	0,00007
Enterococcus	0	0	0	0	0	0	0	0	0	0,0003	0	0,00137	0,00582	0	0,00023	0
Gracilibacillus	0	0	0	0	0	0	0	0	0,00031	0,0008	0,0002	0,001	0,00239	0,0001	0,00077	0
Staphylococcus	0	0	0	0	0	0	0	0	0,00187	0,0013	0	0,00068	0,00085	0	0	0
Unknown Thermoactinomycetaceae	0	0	0	0	0	0	0	0	0,00187	0,0011	0	0,00137	0	0,0002	0,00208	0
Brevibacterium	0	0	0	0	0	0	0	0	0,00009	0	0	0	0	0	0,00068	0
Leucobacter	0	0	0	0	0	0	0	0	0,00053	0	0	0	0	0	0,00009	0
Propionibacterium	0	0	0	0	0	0	0	0	0,00169	0	0	0	0	0	0	0

Unknown Xenococcaceae	0	0	0	0	0	0	0	0	0,00044	0	0	0	0	0	0	0
Unknown Paenibacillaceae	0	0	0	0	0	0	0	0	0,00213	0,001	0,0002	0,00252	0,0005	0,0002	0,00109	0
Unknown Planococcaceae	0	0	0	0	0	0	0	0	0,00138	0,0013	0,0002	0,00074	0	0	0,00005	0
Jeotgalicoccus	0	0	0	0	0	0	0	0	0,00049	0	0	0	0	0	0	0
Unknown Pasteurellales	0	0	0	0	0	0	0	0	0,00209	0	0	0	0	0	0	0
Ureibacillus	0	0	0	0	0	0	0	0	0	0,0014	0	0	0	0	0,00023	0
Aerococcus	0	0	0	0	0	0	0	0	0	0,0003	0	0	0,0002	0	0	0
Bradyrhizobium	0	0	0	0	0	0	0	0	0	0,0001	0	0,00063	0	0	0	0
Unknown Vibrionales	0	0	0	0	0	0	0	0	0	0,0005	0	0	0	0	0	0
Micrococcus	0	0	0	0	0	0	0	0	0	0	0	0,00341	0,00707	0	0	0
Chryseomicrobium	0	0	0	0	0	0	0	0	0	0	0	0,00074	0	0	0	0
Massilia	0	0	0	0	0	0	0	0	0	0	0	0,00053	0	0	0	0
Unknown [Exiguobacteraceae]	0	0	0	0	0	0	0	0	0	0	0	0	0,00244	0	0	0
Unknown Bradyrhizobiaceae	0	0	0	0	0	0	0	0	0	0	0	0	0,00179	0	0	0
Unknown Trebouxiophyceae	0	0	0	0	0	0	0	0	0	0	0	0	0	0,0079	0	0
Cetobacterium	0	0	0	0	0	0	0	0	0	0	0	0	0	0,3209	0	0,00014
Unknown Legionellaceae	0	0	0	0	0	0	0	0	0	0	0	0	0	0,0009	0	0
Guggenheimella	0	0	0	0	0	0	0	0	0	0	0	0	0	0	0,00272	0
Peptoniphilus	0	0	0	0	0	0	0	0	0	0	0	0	0	0	0,00045	0
