# Supplementary material for: Intestinal microbial communities of rainbow trout (Oncorhynchus mykiss) may be improved by feeding a Hermetia illucens meal/low-fishmeal diet
Source: Fish Physiol Biochem. 2021 Jan 3;47(2):365–80. doi: 10.1007/s10695-020-00918-1 (PMC8026480; doi:10.1007/s10695-020-00918-1)
Supplement: Supplementary file 5 — (PDF 232 kb) [file 10695_2020_918_MOESM5_ESM.pdf]

Fish Physiology and Biochemistry

Taxonomic and functional characterization of intestinal microbial communities of rainbow trout (*Oncorhynchus mykiss*) fed with Hermetia illucens meal as alternative protein source.

Simona Rimoldi, Micaela Antonini, Laura Gasco, Federico Moroni, and Genciana Terova. Department of Biotechnology and Life Sciences, University of Insubria, Via J.H. Dunant, 3, 21100 Varese, Italy. genciana.terova@uninsubria.it

Supplementary data file 3. List of predicted functional metagenomics pathways of gut microbiome of trout, as identified by PICRUSt. The mean relative frequency and standard deviation have been reported. The data were analized with STAMP software.

Predicted metagenomic pathways	Ctrl: mean rel. freq. (%)	std. dev. (%)	Hi15: mean rel. freq. (%)	std. dev. (%)	p-values
ABC transporters	4,3896	0,5337	4,6884	0,6959	0,3836
Alanine, aspartate and glutamate metabolism	0,6650	0,0642	0,6775	0,0214	0,6377
Amino acid metabolism	0,0988	0,0382	0,1058	0,0449	0,7592
Amino acid related enzymes	0,0466	0,0195	0,0477	0,0232	0,9218
Amino sugar and nucleotide sugar metabolism	0,8850	0,1206	0,8789	0,0743	0,9106
Aminoacyl-tRNA biosynthesis	1,6545	0,3196	1,7561	0,2697	0,5312
Aminobenzoate degradation	0,0274	0,0254	0,0363	0,0269	0,5361
Amoebiasis	0,0000	0,0000	0,0021	0,0015	0,0071
Antimicrobial resistance genes	0,0259	0,0331	0,0270	0,0189	0,9428
Apoptosis	0,0081	0,0115	0,0003	0,0004	0,1145
Apoptosis - fly	0,0862	0,0101	0,0691	0,0169	0,0412
Arabinogalactan biosynthesis - Mycobacterium	0,0326	0,0249	0,0534	0,0277	0,1629
Arachidonic acid metabolism	0,0236	0,0229	0,0191	0,0114	0,6496
Arginine and proline metabolism	0,6717	0,0717	0,6256	0,0509	0,1902
Arginine biosynthesis	0,3111	0,0365	0,3235	0,0475	0,5938
Ascorbate and aldarate metabolism	0,0417	0,0371	0,0183	0,0087	0,1441
Autophagy - yeast	0,0045	0,0038	0,0140	0,0042	0,0006
Bacterial chemotaxis	0,1498	0,1417	0,0997	0,0544	0,4049
Bacterial invasion of epithelial cells	0,0001	0,0001	0,0006	0,0005	0,0242
Bacterial motility proteins	0,0933	0,0932	0,0813	0,0443	0,7650
Bacterial secretion system	0,3326	0,3412	0,0298	0,0477	0,0516
Bacterial toxins	0,0521	0,0289	0,0533	0,0265	0,9362
Basal transcription factors	0,0010	0,0018	0,0038	0,0033	0,0677
Base excision repair	0,4536	0,0702	0,4831	0,0582	0,4057

Benzoate degradation	0,0632	0,0522	0,0701	0,0543	0,8132
beta-Alanine metabolism	0,0567	0,0174	0,0604	0,0264	0,7569
beta-Lactam resistance	0,1578	0,1211	0,0465	0,0199	0,0457
Betalain biosynthesis	0,0435	0,0194	0,0366	0,0175	0,4968
Biofilm formation - Escherichia coli	0,0882	0,0904	0,0071	0,0070	0,0494
Biofilm formation - Pseudomonas aeruginosa	0,0666	0,0771	0,0035	0,0063	0,0673
Biofilm formation - Vibrio cholerae	0,1887	0,0941	0,1190	0,0565	0,1199
Biosynthesis of siderophore group nonribosomal peptides	0,0308	0,0455	0,0010	0,0012	0,1269
Biosynthesis of unsaturated fatty acids	0,1332	0,0605	0,1040	0,0499	0,3413
Biosynthesis of various secondary metabolites - part 2	0,0000	0,0001	0,0000	0,0000	0,5387
Biotin metabolism	0,2565	0,0571	0,2213	0,0744	0,3381
Butanoate metabolism	0,4695	0,1156	0,5081	0,1092	0,5312
C5-Branched dibasic acid metabolism	0,1431	0,0495	0,1522	0,0642	0,7729
Caprolactam degradation	0,0008	0,0013	0,0012	0,0007	0,5709
Carbohydrate metabolism	0,0633	0,0273	0,0943	0,0470	0,1599
Carbon fixation in photosynthetic organisms	0,0663	0,0480	0,0674	0,0338	0,9630
Carbon fixation pathways in prokaryotes	0,1574	0,0236	0,1585	0,0276	0,9346
Carotenoid biosynthesis	0,1537	0,1248	0,1737	0,0809	0,7290
Cationic antimicrobial peptide (CAMP) resistance	0,1885	0,0621	0,2265	0,0597	0,2633
Cell cycle - Caulobacter	0,6331	0,0781	0,6490	0,0466	0,6524
Cell growth	0,1413	0,0125	0,9064	0,5181	0,0058
Cell motility	0,0384	0,0402	0,0060	0,0037	0,0703
Chagas disease (American trypanosomiasis)	0,0169	0,0223	0,0006	0,0006	0,0956
Chaperones and folding catalysts	1,0713	0,1528	0,8721	0,0278	0,0105
Chloroalkane and chloroalkene degradation	0,0058	0,0047	0,0087	0,0038	0,2307
Chlorocyclohexane and chlorobenzene degradation	0,0000	0,0000	0,0001	0,0001	0,1284
Cholinergic synapse	0,0000	0,0000	0,0000	0,0000	0,1067
Chromosome and associated proteins	1,3557	0,1895	1,3018	0,1425	0,5580
Citrate cycle (TCA cycle)	0,5557	0,1237	0,5294	0,1523	0,7277
Cofactor metabolism	0,1687	0,0346	0,1601	0,0225	0,5913
Cysteine and methionine metabolism	0,9299	0,0724	0,9595	0,0229	0,3312
Cytochrome P450	0,0005	0,0005	0,0101	0,0070	0,0086
Cytoskeleton proteins	0,0087	0,0109	0,0104	0,0072	0,7300

D-Alanine metabolism	0,2367	0,0625	0,2460	0,0693	0,7947
D-Arginine and D-ornithine metabolism	0,0002	0,0003	0,0003	0,0003	0,6753
D-Glutamine and D-glutamate metabolism	0,2209	0,0428	0,2366	0,0376	0,4792
Dioxin degradation	0,0002	0,0002	0,0002	0,0002	0,9889
DNA repair and recombination proteins	0,9416	0,0922	0,9855	0,0718	0,3380
DNA replication	1,0334	0,2175	1,0757	0,2153	0,7199
DNA replication proteins	0,5578	0,1145	0,5354	0,1176	0,7237
Drug metabolism - other enzymes	0,0078	0,0113	0,0001	0,0002	0,1188
Endocytosis	0,0000	0,0000	0,0000	0,0000	0,0307
Energy metabolism	0,4369	0,2507	0,2891	0,0892	0,1768
Enzymes with EC numbers	3,7158	0,2349	3,6314	0,1696	0,4554
Epithelial cell signaling in Helicobacter pylori infection	0,0761	0,0329	0,0566	0,0279	0,2510
Exosome	0,1103	0,0238	0,1042	0,0173	0,5916
Fatty acid biosynthesis	0,8253	0,0860	0,8595	0,0739	0,4372
Fatty acid degradation	0,0423	0,0394	0,0416	0,0255	0,9694
Flagellar assembly	0,4592	0,4254	0,3005	0,1599	0,3797
Fluorobenzoate degradation	0,0425	0,0265	0,0355	0,0162	0,5613
Folate biosynthesis	0,9995	0,1185	0,9813	0,1262	0,7854
Fructose and mannose metabolism	0,4744	0,0691	0,5414	0,0405	0,0482
Function unknown	4,0264	0,3093	3,7808	0,1299	0,0833
Galactose metabolism	0,4809	0,2106	0,5559	0,2339	0,5388
General function prediction only	1,4203	0,4016	1,2607	0,2951	0,4123
Geraniol degradation	0,0004	0,0005	0,0001	0,0001	0,2621
Global maps only	0,0171	0,0142	0,0119	0,0065	0,4002
Glutathione metabolism	0,4273	0,1044	0,3599	0,0749	0,1889
Glycan metabolism	0,0749	0,0194	0,0656	0,0163	0,3466
Glycerolipid metabolism	0,5299	0,0893	0,5464	0,1116	0,7653
Glycerophospholipid metabolism	0,4810	0,1092	0,4601	0,0716	0,6791
Glycine, serine and threonine metabolism	0,6764	0,0424	0,6980	0,0674	0,4879
Glycolysis / Gluconeogenesis	2,1433	0,3014	2,2583	0,2854	0,4756
Glycosyltransferases	0,0964	0,0707	0,0756	0,0962	0,6530
Glyoxylate and dicarboxylate metabolism	0,8100	0,1509	0,8420	0,2027	0,7431
Histidine metabolism	0,5836	0,1079	0,5949	0,1488	0,8741

Homologous recombination	0,7095	0,1009	0,6974	0,1057	0,8296
Inositol phosphate metabolism	0,1098	0,0266	0,1191	0,0170	0,4536
Insulin signaling pathway	0,0003	0,0002	0,0102	0,0071	0,0075
Isoflavonoid biosynthesis	0,0075	0,0114	0,0002	0,0002	0,1355
Legionellosis	0,0736	0,0144	0,0770	0,0118	0,6388
Linoleic acid metabolism	0,0005	0,0008	0,0001	0,0002	0,3091
Lipid biosynthesis proteins	0,0167	0,0162	0,0316	0,0202	0,1507
Lipid metabolism	0,0010	0,0015	0,0002	0,0001	0,1753
Lipoarabinomannan (LAM) biosynthesis	0,0001	0,0001	0,0003	0,0003	0,2222
Lipoic acid metabolism	0,1866	0,0623	0,2147	0,0616	0,4095
Lipopolysaccharide biosynthesis	0,5546	0,2464	0,3445	0,2212	0,1157
Lipopolysaccharide biosynthesis proteins	0,0157	0,0178	0,0014	0,0012	0,0717
Longevity regulating pathway - multiple species	0,0773	0,0414	0,0803	0,0332	0,8829
Lysine biosynthesis	0,8668	0,3567	0,8770	0,4450	0,9631
Lysine degradation	0,0273	0,0340	0,0438	0,0558	0,5177
MAPK signaling pathway - fly	0,0838	0,0406	0,0871	0,0402	0,8812
MAPK signaling pathway - plant	0,0741	0,0144	0,0846	0,0074	0,1163
Membrane trafficking	0,0655	0,0485	0,0713	0,0358	0,8031
Messenger RNA biogenesis	0,1441	0,0842	0,1228	0,0772	0,6298
Methane metabolism	0,3188	0,0833	0,3147	0,0451	0,9126
Mineral absorption	0,0362	0,0229	0,0534	0,0264	0,2149
Mismatch repair	0,4415	0,0719	0,4538	0,0309	0,6856
Mitochondrial biogenesis	0,0622	0,0215	0,0570	0,0245	0,6760
Monobactam biosynthesis	0,0002	0,0003	0,0001	0,0001	0,2968
N-Glycan biosynthesis	0,0030	0,0044	0,0025	0,0039	0,8089
Naphthalene degradation	0,0001	0,0002	0,0001	0,0000	0,5469
Necroptosis	0,0167	0,0146	0,0016	0,0018	0,0299
Nicotinate and nicotinamide metabolism	0,6797	0,0849	0,6761	0,0337	0,9200
Nitrogen metabolism	0,5375	0,1651	0,4902	0,1735	0,6095
Nitrotoluene degradation	0,0633	0,0400	0,0368	0,0179	0,1423
NOD-like receptor signaling pathway	0,1305	0,0385	0,1416	0,0280	0,5461
Non-homologous end-joining	0,0005	0,0007	0,0223	0,0153	0,0072
Nonribosomal peptide structures	0,0290	0,0259	0,0349	0,0177	0,6270

Nucleotide excision repair	0,4336	0,1032	0,4864	0,0667	0,2780
Nucleotide metabolism	0,0568	0,0539	0,0574	0,0685	0,9844
One carbon pool by folate	0,1459	0,0269	0,1538	0,0236	0,5685
Other glycan degradation	0,0021	0,0023	0,0368	0,0255	0,0085
Others	0,4968	0,1580	0,4123	0,1412	0,3095
Oxidative phosphorylation	2,0724	0,3766	2,0479	0,3527	0,9017
Pantothenate and CoA biosynthesis	0,4443	0,0790	0,4768	0,0589	0,3986
Parathyroid hormone synthesis, secretion and action	0,0076	0,0114	0,0001	0,0002	0,1257
Penicillin and cephalosporin biosynthesis	0,0319	0,0260	0,0548	0,0245	0,1123
Pentose and glucuronate interconversions	0,2343	0,1011	0,2709	0,0873	0,4812
Pentose phosphate pathway	1,0099	0,2384	1,1061	0,2113	0,4376
Peptidases and inhibitors	1,9402	0,1471	1,9687	0,1533	0,7276
Peptidoglycan biosynthesis	0,8404	0,1321	0,9291	0,0832	0,1590
Peptidoglycan biosynthesis and degradation proteins	0,2291	0,1058	0,1247	0,0527	0,0409
Peroxisome	0,0418	0,0255	0,0521	0,0262	0,4665
Pertussis	0,0048	0,0093	0,0007	0,0007	0,2820
Phenazine biosynthesis	0,0005	0,0008	0,0001	0,0001	0,1920
Phenylalanine metabolism	0,0992	0,0511	0,0720	0,0329	0,2599
Phenylalanine, tyrosine and tryptophan biosynthesis	0,8896	0,1276	0,9207	0,1240	0,6503
Phenylpropanoid biosynthesis	0,0002	0,0003	0,0037	0,0094	0,3625
Phosphonate and phosphinate metabolism	0,1012	0,0409	0,0795	0,0107	0,2111
Phosphotransferase system (PTS)	0,1388	0,0801	0,1027	0,0803	0,4129
Photosynthesis	1,1029	0,9856	1,2767	0,6384	0,7022
Photosynthesis - antenna proteins	0,3482	0,3113	0,4031	0,2017	0,7026
Photosynthesis proteins	0,0438	0,0194	0,0450	0,0221	0,9152
Plant-pathogen interaction	0,1036	0,0253	0,0796	0,0124	0,0480
Polyketide biosynthesis proteins	0,0003	0,0004	0,0158	0,0111	0,0076
Polyketide sugar unit biosynthesis	0,2214	0,0467	0,2427	0,0597	0,4703
Porphyrin and chlorophyll metabolism	1,5429	0,8262	1,7054	0,7155	0,7002
Primary bile acid biosynthesis	0,0526	0,0499	0,0529	0,0632	0,9927
Prokaryotic defense system	0,7478	0,1872	0,7819	0,0646	0,6597
Propanoate metabolism	0,3104	0,0927	0,2745	0,0131	0,3427
Proteasome	0,0001	0,0002	0,0008	0,0012	0,1407

Protein digestion and absorption	0,0006	0,0009	0,0000	0,0000	0,1427
Protein export	1,0538	0,1121	1,0993	0,0458	0,3448
Protein kinases	0,5122	0,2597	0,5529	0,2064	0,7505
Protein phosphatases and associated proteins	0,0001	0,0001	0,0002	0,0002	0,1452
Protein processing	0,6698	0,1326	0,6352	0,1117	0,6061
Protein processing in endoplasmic reticulum	0,1048	0,0312	0,1241	0,0104	0,1562
Purine metabolism	2,8203	0,4320	2,8229	0,4891	0,9917
Pyrimidine metabolism	1,5631	0,2941	1,6276	0,3122	0,6970
Pyruvate metabolism	1,1608	0,1431	1,1624	0,1809	0,9861
Quorum sensing	0,5720	0,2500	0,7471	0,3337	0,2867
Renin-angiotensin system	0,0359	0,0230	0,0343	0,0165	0,8844
Replication and repair	0,8102	0,1495	0,7310	0,1384	0,3216
Riboflavin metabolism	0,3320	0,0423	0,3259	0,0540	0,8171
Ribosome	3,9508	0,7823	4,1230	0,6861	0,6683
Ribosome biogenesis	2,6831	0,3492	2,5778	0,4271	0,6218
Ribosome biogenesis in eukaryotes	0,1249	0,0131	0,1121	0,0075	0,0453
RNA degradation	0,7964	0,0766	0,8089	0,0248	0,6931
RNA polymerase	0,3477	0,0777	0,3790	0,0578	0,4077
RNA transport	0,1957	0,0673	0,2190	0,0190	0,4049
Secondary bile acid biosynthesis	0,0479	0,0174	0,0549	0,0226	0,5255
Secondary metabolism	0,0169	0,0188	0,0019	0,0015	0,0723
Secretion system	1,0411	0,2839	0,7875	0,0989	0,0536
Selenocompound metabolism	0,3317	0,0644	0,3825	0,0619	0,1541
Sesquiterpenoid and triterpenoid biosynthesis	0,0293	0,0258	0,0336	0,0168	0,7158
Shigellosis	0,0028	0,0063	0,0003	0,0003	0,3229
Signaling proteins	0,3830	0,0557	0,3168	0,0207	0,0165
Sphingolipid metabolism	0,0092	0,0114	0,0035	0,0024	0,2311
Staphylococcus aureus infection	0,0003	0,0003	0,0020	0,0016	0,0281
Starch and sucrose metabolism	0,9364	0,1247	1,0636	0,0549	0,0339
Staurosporine biosynthesis	0,0002	0,0002	0,0001	0,0001	0,3508
Steroid biosynthesis	0,0290	0,0259	0,0394	0,0200	0,4154
Steroid degradation	0,0000	0,0001	0,0006	0,0004	0,0080
Steroid hormone biosynthesis	0,00 €	0,00 €	0,00 €	0,00 €	0,01 €

Structural proteins	0,3349	0,2079	0,1671	0,0709	0,0755
Sulfur metabolism	0,8700	0,1925	0,9359	0,2095	0,5501
Sulfur relay system	0,2573	0,0611	0,2191	0,0504	0,2241
Taurine and hypotaurine metabolism	0,0394	0,0232	0,0445	0,0228	0,6818
Terpenoid backbone biosynthesis	0,5995	0,1088	0,6380	0,0827	0,4692
Thiamine metabolism	0,7739	0,1243	0,8187	0,1200	0,5041
Toll and Imd signaling pathway	0,0000	0,0000	0,0003	0,0002	0,0245
Toxoplasmosis	0,0000	0,0000	0,0001	0,0000	0,0135
Transcription	0,2408	0,0943	0,2342	0,0830	0,8915
Transcription factors	1,9550	0,6549	1,9385	0,3971	0,9555
Transcription machinery	0,7811	0,0328	0,8908	0,0943	0,0181
Transfer RNA biogenesis	2,0173	0,1175	1,9304	0,0732	0,1233
Translation	0,0306	0,0292	0,0478	0,0232	0,2443
Translation factors	1,0237	0,1595	1,0473	0,1370	0,7717
Transport	0,8765	0,1964	0,9104	0,2750	0,7951
Transporters	7,4576	0,4918	7,5905	0,2620	0,5414
Tropane, piperidine and pyridine alkaloid biosynthesis	0,0001	0,0001	0,0060	0,0042	0,0072
Tryptophan metabolism	0,0109	0,0116	0,0137	0,0093	0,6348
Two-component system	2,5990	0,8253	2,2378	0,2502	0,2989
Tyrosine metabolism	0,1219	0,0328	0,1132	0,0383	0,6550
Ubiquinone and other terpenoid-quinone biosynthesis	0,6375	0,1725	0,5888	0,1773	0,6111
Ubiquitin system	0,0000	0,0000	0,0002	0,0002	0,1533
Valine, leucine and isoleucine biosynthesis	0,1396	0,0519	0,1387	0,0577	0,9770
Valine, leucine and isoleucine degradation	0,0987	0,0417	0,0804	0,0135	0,3019
Vibrio cholerae infection	0,0136	0,0149	0,0006	0,0013	0,0555
Vitamin B6 metabolism	0,1923	0,0447	0,1834	0,0357	0,6873
Yersinia infection	0,0054	0,0141	0,0002	0,0002	0,3597
Zeatin biosynthesis	0,0736	0,0144	0,0770	0,0118	0,6410
